# Factors Influencing the Adoption of Contact Tracing Applications: Systematic Review and Recommendations

**DOI:** 10.3389/fdgth.2022.862466

**Published:** 2022-05-03

**Authors:** Kiemute Oyibo, Kirti Sundar Sahu, Arlene Oetomo, Plinio Pelegrini Morita

**Affiliations:** ^1^School of Public Health Sciences, University of Waterloo, Waterloo, ON, Canada; ^2^Institute of Health Policy, Management, and Evaluation, University of Toronto, Toronto, ON, Canada; ^3^Department of Systems Design Engineering, University of Waterloo, Waterloo, ON, Canada; ^4^eHealth Innovation, Techna Institute, University Health Network, Toronto, ON, Canada; ^5^Research Institute for Aging, University of Waterloo, Waterloo, ON, Canada

**Keywords:** COVID-19, contact tracing app, adoption, facilitators, barriers

## Abstract

**Background:**

The emergence of new variants of COVID-19 causing breakthrough infections and the endemic potential of the coronavirus are an indication that digital contact tracing apps (CTAs) may continue to be useful for the long haul. However, the uptake of these apps in many countries around the world has been low due to several factors militating against their adoption and usage.

**Objective:**

In this systematic review, we set out to uncover the key factors that facilitate or militate against the adoption of CTAs, which researchers, designers and other stakeholders should focus on in future iterations to increase their adoption and effectiveness in curbing the spread of COVID-19.

**Data Sources:**

Seven databases, including PubMed, CINAHL, Scopus, Web of Service, IEEE Xplore, ACM Digital Library, and Google Scholar, were searched between October 30 and January 31, 2020. A total of 777 articles were retrieved from the databases, with 13 of them included in the systematic review after screening.

**Study Eligibility Criteria, Participants, and Intervention:**

The criteria for including articles in the systematic review were that they could be user studies from any country around the world, must be peer-reviewed, written in English, and focused on the perception and adoption of COVID-19 contact tracing and/or exposure notification apps. Other criteria included user study design could be quantitative, qualitative, or mixed, and must have been conducted during the COVID-19 pandemic, which began in the early part of 2020.

**Study Appraisal and Synthesis Methods:**

Three researchers searched seven databases (three by the first author, and two each by the second and third authors) and stored the retrieved articles in a collaborative Mendeley reference management system online. After the removal of duplicates, each researcher independently screened one third of the articles based on title/abstract. Thereafter, all three researchers collectively screened articles that were in the borderline prior to undergoing a full-text review. Then, each of the three researchers conducted a full-text review of one-third of the eligible articles to decide the final articles to be included in the systematic review. Next, all three researchers went through the full text of each borderline article to determine their appropriateness and relevance. Finally, each researcher extracted the required data from one-third of the included articles into a collaborative Google spreadsheet and the first author utilized the data to write the review.

**Results:**

This review identified 13 relevant articles, which found 56 factors that may positively or negatively impact the adoption of CTAs. The identified factors were thematically grouped into ten categories: privacy and trust, app utility, facilitating conditions, social-cognitive factors, ethical concerns, perceived technology threats, perceived health threats, technology familiarity, persuasive design, and socio-demographic factors. Of the 56 factors, privacy concern turned out to be the most frequent factor of CTA adoption (12/13), followed by perceived benefit (7/13), perceived trust (6/13), and perceived data security risk (6/13). In the structural equation models presented by the authors of the included articles, a subset of the 56 elicited factors (e.g., perceived benefit and privacy concern) explains 16 to 77% of the variance of users' intention to download, install, or use CTAs to curb the spread of COVID-19. Potential adoption rates of CTA range from 19% (in Australia) to 75% (in France, Italy, Germany, United Kingdom, and United States). Moreover, actual adoption rates range from 37% (in Australia) to 50% (in Germany). Finally, most of the studies were carried out in Europe (66.7%), followed by North America (13.3%), and Australia, Asia, and South America (6.7% each).

**Conclusion:**

The results suggest that future CTA iterations should give priority to privacy protection through minimal data collection and transparency, improving contact tracing benefits (personal and social), and fostering trust through laudable gestures such as delegating contact tracing to public health authorities, making source code publicly available and stating who will access user data, when, how, and what it will be used for. Moreover, the results suggest that data security and tailored persuasive design, involving reward, self-monitoring, and social-location monitoring features, have the potential of improving CTA adoption. Hence, in addition to addressing issues relating to utility, privacy, trust, and data security, we recommend the integration of persuasive features into future designs of CTAs to improve their motivational appeal, adoption, and the user experience.

**Systematic Review Registration:**

https://www.crd.york.ac.uk/prospero/display_record.php?ID=CRD42021259080 PROSPERO, identifier CRD42021259080.

## Introduction

### Rationale

The COVID-19 pandemic has caused over five million deaths worldwide (1). The health and socio-economic ramifications of the pandemic led most national governments worldwide, especially in the West, to roll out digital contact tracing apps (CTAs) to combat the spread of the virus and augment the traditional manual contact tracing techniques. The emergence of new variants of COVID-19 such as the Delta and Omicron variants, which can cause breakthrough infections, and their endemic potential are an indication that CTAs may continue to be useful in our everyday life for a very long time, even after the pandemic (2). Research shows that for CTAs to be effective in containing the virus, a large number of people in the population (e.g., at the national level) ought to adopt and use them regularly. For example, it has been estimated that roughly 60% of the population may need to use CTAs in order for them to be effective in containing the spread of the disease (3). However, current research shows that so far the adoption rate of CTAs has been low due to a number of barriers cutting across so many areas including personal, social, legal, ethical, and technological (4, 5). For example, in Canada, as of November 26, 2020, only about 15% of Canadian residents had downloaded the national CTA called COVID Alert (6). As of mid-2020, in European countries such as France, Italy and Germany, <15% of the population had downloaded their national CTAs. Moreover, in Asian countries such as Thailand, Vietnam, and Philippines, <1% of the population had downloaded their national CTAs (5). Hence, it becomes important for researchers to investigate not only the barriers but the facilitators as well so that designers and sponsors of CTAs will be well informed about what specific factors they ought to focus on in the design of future CTAs to make them more acceptable and effective. Particularly, a systematic review of the existing studies on technology acceptance and adoption becomes pertinent. Hence, our systematic review is timely as most of existing literature only reported a subset of the facilitators and barriers associated with CTA adoption (3). According to Zhang et al. (3), “*There is a dearth of evidence [especially systematic reviews] regarding the barriers and facilitators to uptake and engagement with COVID-19 digital contact tracing applications*.” Although a handful of systematic reviews have been carried out, the majority of them were not related to technology acceptance, Moreover, most of the reviews were carried out at the early stage of the pandemic in 2020 when only few empirical studies had been conducted, peer-reviewed, and published. Specifically, systematic reviews such as Braithwaite et al. (7), Davalbhakta et al. (8) and Juneau et al. (9) were not particularly focused on technology acceptance aimed to uncover the facilitators and barriers militating against CTA adoption.

Hence, we set out to conduct a systematic review of the empirical studies carried out so far. The review is aimed at: (1) investigating users' perceptions, the facilitators and barriers associated with the adoption of CTAs, moderators, and motivational strategies, if any; (2) recommending design guidelines; (3) and identifying opportunities for future research.

### Objective

The systematic review was aimed at identifying user studies focused on user perception and technology acceptance of CTAs and eliciting the factors (facilitators and barriers) affecting their adoption. Specifically, the review set out to address the overarching research question, “*How are Covid-19 CTAs perceived by the general population?*” This research question is broken down into the following three subquestions:

RQ1: What are the key facilitators and barriers that are associated with the adoption of contact tracing apps?
RQ2: What motivational strategies are being implemented to increase the adoption of contact tracing apps?
RQ3: What are the adoption rates of contact tracing apps among their target audiences?

### Protocol and Registration

The protocol for the systematic review was published in the Journal of Medical Internet Research (JMIR) on June 1, 2021 (10). Moreover, the systematic review was registered with National Institute for Health Research (NIHR) systematic review database (PROSPERO) on December 8, 2021. The registration number is CRD42021259080.

## Methods

### Eligibility Criteria

To be included in the review, the article: (1) must be an empirical study that evaluated users' perceptions and technology acceptance of COVID-19 CTAs; (2) can be from any country around the world; (3) can have a quantitative, qualitative, or mixed-method study design; and (4) must be written in English and peer-reviewed; (5) must focus on factors (facilitators and barriers) that influence CTA acceptance, adoption and/or uptake.

### Information Sources

Three of the authors searched seven databases focused on health and technology, which include PubMed, CINAHL, Scopus, Web of Service, ACM Digital Library, IEEE Xplore, and Google Scholar. For example, PubMed is a health-based search engine for accessing and retrieving MEDLINE database of articles related to life sciences and biomedical topics. Moreover ACM Digital Library and IEEE Xplore are technology-based databases for accessing technical articles dealing with computer science and engineering.

### Search

The first three authors searched the seven databases, with the first author handling three databases, and the second and third authors handling two databases each. Each of the databases were searched using the terms related to contact tracing, technology, adoption, and COVID-19. The whole set of keywords used in searching the databases (except for Google Scholar) includes: (“contact tracing” OR “contact-tracing” OR “exposure notification” OR “exposure-notification” OR “contact notification” OR “contact-notification” OR “GAEN”) AND (app OR apps OR application^*^ OR technolog^*^ OR system OR systems) AND (percept^*^ OR adopt^*^ OR accept^*^ OR uptake OR use OR usage) AND (covid^*^ OR coronavirus OR SARS-CoV-2). The criterion for the search is “ALL,” meaning articles were searched based on title, abstract, keyword, full text, etc.

### Selection of Sources of Evidence

The articles retrieved from the databases were automatically stored in our collaborative Mendeley reference management system using the Mendeley Web Importer, a web-browser extension that helps researchers to import references (full-text PDF documents) into reference management systems. All of the articles from the different databases were combined in Mendeley, after which they were exported to a Google spreadsheet to remove duplicates. The first three authors underwent the screening, selection, and full-text review of the articles retrieved from the databases. After the removal of duplicates, each author screened an approximately equal number of articles based on titles and/or abstracts and excluded those that did not meet the eligibility criteria. Articles that were in question were labeled “maybe.” All three authors collaborated to determine the eligibility of the “maybe” articles. Afterwards, a full-text review was carried out on the articles that met the inclusion criteria. Finally, based on the inclusion criteria, all three authors reviewed the included articles collaboratively to confirm and validate them.

### Data Collection

After screening and selecting the final number of articles included in the systematic review, each of the three authors went through one-third of the total articles to extract the important themes of interests and their corresponding values. These themes and their values were tabulated in an Google spreadsheet, which allowed all three authors to collaborate and discuss the results.

### Data Items

Twelve themes (characteristics) were elicited from the relevant articles. Table 1 shows all 12 themes and their descriptions/examples. The themes include author names, study date, target audience, facilitators, barriers, outcome variables, recommendations, and opportunities for future studies.

**Table 1:** Analysis coding scheme for systematic review.

**S/N**	**Characteristics**	**Description/example**
1	Authors	Name of authors
2	Study date	Month and year study was carried out
3	Type of application	Description based, prototype
4	Target audience	Country, sample size, age
5	Type of study	Quantitative, qualitative, mixed
6	Outcome variable	Intention to download app, intention to install app, intention to use app, etc.
7	Facilitators	Perceived usefulness, perceived trust, etc.
8	Barriers	Privacy concern, perceived risk, etc.
9	Moderating variables	Socio-demographic variables such as Age, gender, culture, etc.
10	Findings/Takeaways	Summary of the main findings and takeaways
11	Recommendations	Proposed guidelines for effective design of CTAs

### Synthesis Methods

We synthesized the results from the 13 articles included in the review using graphical, visual, tabulation, and narrative approaches (11). The graphical approach enabled us to summarize our findings in terms of continent of study, type of study, and number of participants. The visual approach enabled us to utilize a fishbone diagram to summarize the significant factors that influence CTA adoption under 10 thematic categories. A fishbone diagram, sometimes called cause and effect diagram, is a visual tool that helps in “*brainstorming to identify possible causes of a problem and in sorting ideas into useful categories*” (p. 1) (12). Moreover, the tabular approach enabled us to classify the elicited factors of CTA adoption as facilitators and barriers in each thematic category in a table we called the factors table. Particularly, the factors table enabled us to indicate and count the number of articles associated with each factor in a category. The first author employed affinity diagramming method (13) to organize the factors elicited from the included articles into the thematic categories in the fishbone diagram; thereafter, the categorization was discussed and refined by all of the authors. Finally, we used the narrative approach to discuss our findings and their implications for CTA design in the future.

## Results

In this section, we present the results of the article selection process, the key characteristics of the articles included in the systematic review, the articles' risk of bias, and the results of the articles based on the three main research questions.

### Study Selection

**Figure 1:**
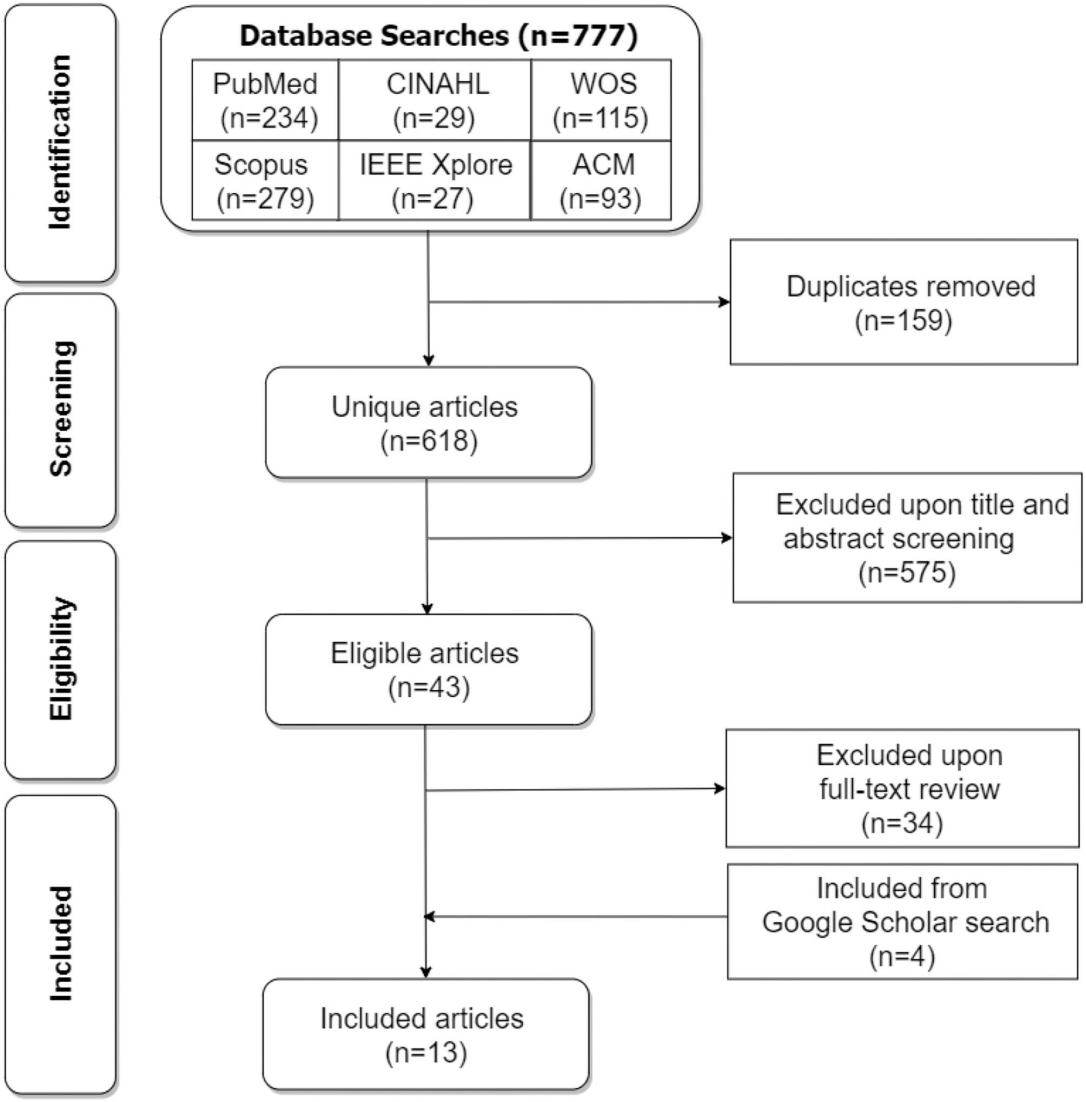
PRISMA flowchart for the screening and inclusion of articles in the review (WOS, Web of Science, ACM, Association for Computing Machinery).

We utilized the PRISMA [Preferred Reporting Items for Systematic Review and Meta-analysis (14)] flowchart shown in Figure 1 to identify, screen, and select the relevant articles to be included in the systematic review. Two approaches were used in searching for articles in seven databases: formal and informal. In the formal approach, we searched six databases (PubMed, CINAHL, Web of Science, Scopus, IEEE Xplore, and ACM Digital Library) systematically, and retrieved 777 articles in total between October 30, 2020, and November 20, 2020. In the screening phase, we removed 159 duplicates to arrive at 618 articles. In the eligibility phase, we screened out 575 articles to arrive at 43 articles. In the inclusion phase, we excluded 34 articles upon full-text review to arrive at 9 articles. The informal approach involved Google Scholar. Carried out between November 21, 2020, and January 31, 2021, it was aimed to uncover more articles that might not have been retrieved through the formal search carried out between October 30, 2020, and November 20, 2020. By the end of January 31, 2021, we found 4 additional articles from the Google Scholar search, which we added to the initial 9 articles from the formal search. Altogether, we had 13 articles in total for the final systematic review (10).

### Study Characteristics

Several themes (characteristics) were elicited from each of the 13 articles included in the systematic review and entered into a Google spreadsheet in the data extraction step (10). For each of the 13 articles, the extracted data for the key themes, such as author names, study date, country of study, target audience, facilitators, barriers, outcome variables, and recommendations, are presented in Supplementary Table 1.

### Risk of Bias of Studies

The individual studies presented in Supplementary Table 1 have one risk of bias or another. The first risk of bias is sample size. While some of the studies [e.g., (15, 16)] have relatively large sample sizes over 1,000, others [e.g., (17, 18)] have small sample sizes <300. The studies with larger samples sizes that presented structural equation models (SEMs) are more likely to have statistically significant relationships between constructs, compared with those with smaller sample sizes, due to an increased level of confidence in the presented result which larger sample sizes foster. In other words, studies with larger sample sizes are more likely to produce findings that can generalize to the wider population than those with smaller sample sizes (19). The problem of sample size would have been addressed using the effect size metric rather than the significance level. However, most of the reviewed studies did not compute or report effect size.

The second risk of bias is that the studies are carried out at different times during the COVID-19 pandemic. Approximately, two-third of the included studies [e.g., (15, 16, 20)] were carried out during the first half of 2020 when many were yet to be familiar with CTAs. The other one-third of the studies [e.g., (17, 21, 22)] were carried out in the second half of the same year when people were more familiar with CTAs and their potential benefits due to the marketing campaigns and the availability of more information on traditional and social media. The difference in times in the conduct of the studies may have influenced the various findings. For example, respondents might have been less willing to adopt CTAs due to misconceptions and misinformation (especially relating to government surveillance) at the outset of the pandemic.

The third risk of bias is that the studies applied different research designs and stimuli. For example, while some of the studies were based on a textual description of hypothetical CTAs (22–24), others were based on screenshots of hypothetical prototypes (17, 25), or the description of a national app (26). These differences in stimuli might have influenced the findings in the different studies one way or the other. For example, respondents might have been more favorable to a national app they were familiar with compared with a hypothetical app they were yet to use.

The fourth risk of bias is the type of participants recruited in the individual studies, which may be influenced by the method and medium of recruitment of participants. For example, if a study found that X% of the participants were willing to download a given CTA if it were deployed in real life, this might not reflect the actual percentage of willing adopters in the wider population. The reason is that the participants of the study in question [e.g., recruited on a crowdsourcing platform or social media (24, 27)] might be more computer literate, educated, and well-informed about the utility of CTAs than the wider population. Hence, they may be more likely to adopt CTAs compared with the average person in the general population who may be less informed and aware of the utility of CTAs.

The fifth risk of bias is the method of analysis employed in analyzing the data in the individual studies. While some of the studies [e.g. (15, 20, 27)] employed quantitative analyses, others [e.g., (17, 18, 26)] used mixed-method analyses. Research shows that mixed-method research produces stronger findings as researchers can triangulate the quantitative findings with the qualitative findings. Moreover, among the studies that employed quantitative analyses, different statistical methods were used including SEM (15, 27), multiple regression modeling (23, 25), and logistic regression modeling (20). For example, one major difference between multiple regression and SEM is that the former only deals with the observed variables, while the latter handles both the observed and the unobserved (latent) variables. Moreover, unlike traditional multiple regression, SEM supports the comparison of models across different groups, the inclusion of multiple dependent variables, and the investigation of both mediating and moderating variables (28). These differences between analytical methods have the potential to impact research findings one way or the other.

### Descriptive Statistics

**Figure 2:**
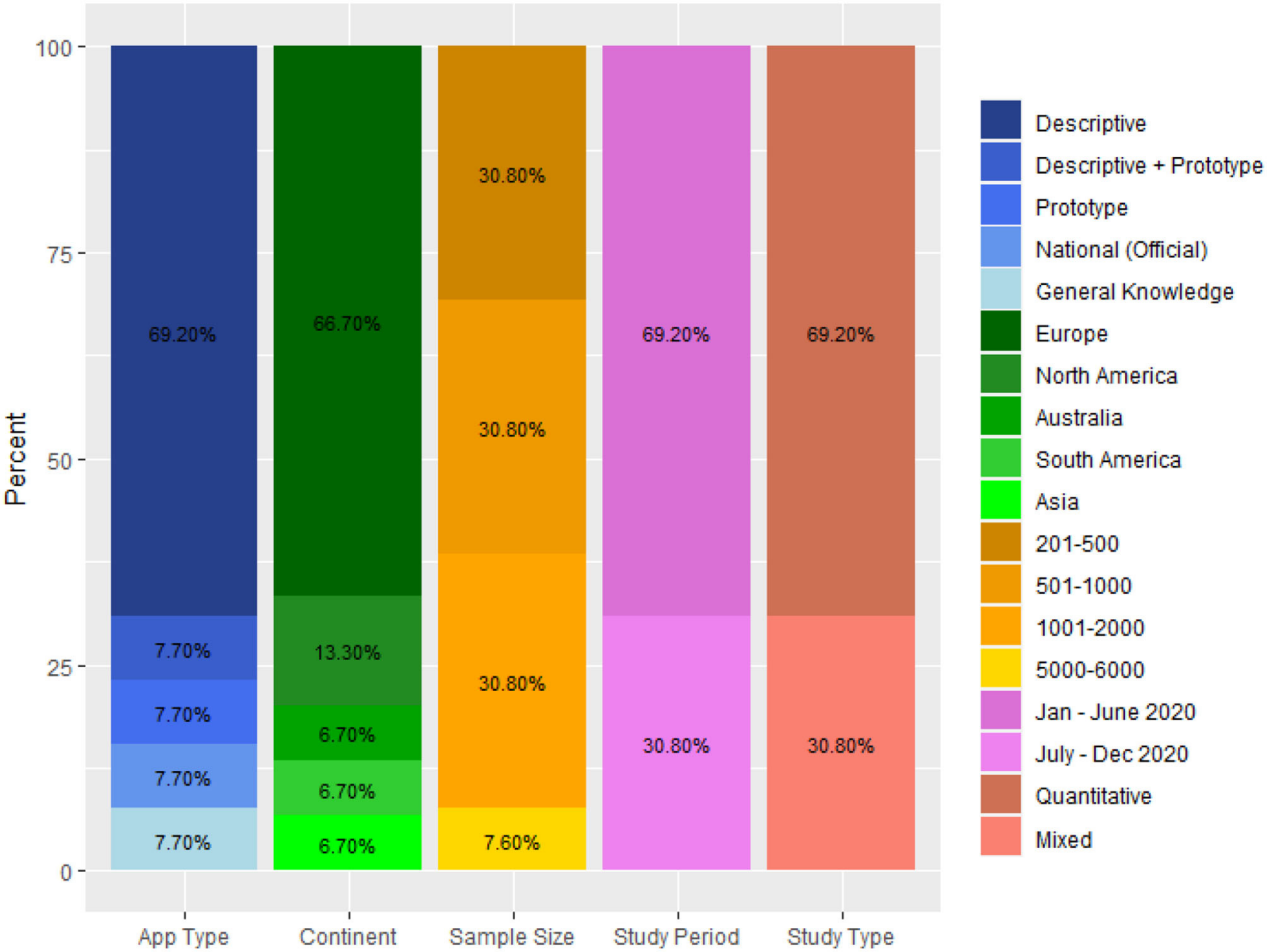
Bar chart showing the distribution of studies in terms of app type, continent of study, sample size, study period, and study type.

Figure 2 shows the distribution of the included studies in terms of app type, continent of study, sample size, study period, and study type. The bar chart is based on the key tabulated data in Supplementary Table 1. Each column of the bar chart represents the distribution of the 13 studies based on each factor. For example, the first column, denoted with a blue color band in the legend on the right, represents the study distribution in terms of app type, which includes descriptive, descriptive and prototype, prototype, national (official), and general knowledge of CTAs. Descriptive, for instance, means that the study was based on the description of a hypothetical CTA having certain functionalities. Of the 13 reviewed studies, nine (69.2%) were based on the description of the functionalities of a CTA, and one each (7.7%) on an official national app, a mocked-up prototype, general knowledge of CTAs, or a combination of app description and prototype. Secondly, most of the studies (two-third or 66.7%) have been carried out among people in Europe; the other one-third (33.3%) among people in North America, Australia, South America, and Asia. Moreover, a larger number of the studies (about two-third or 69.2%) were quantitative; the other one-third (30.8%) were based on a mixed-method approach. Similarly, a larger number of the studies (two-third or 69.2%) were carried out in the first half of 2020, and the other one-third (30.8%) were conducted in the second half. Finally, approximately one-third (30.8%) of the studies employed a sample size that ranged between 201 and 500, or 501 and 1,000, or 1,001 and 2,000.

### Factors of CTA Adoption

The first set of results addresses the first research question, “*What are the key facilitators and barriers that are associated with the adoption of contact tracing apps?*” We elicited 56 factors affecting CTA adoption, which are grouped into 10 thematic categories such as privacy and trust, perceived utility, facilitating conditions, and ethical concerns. Each of the 10 categories (composed of both facilitators and barriers) is defined in Table 2. Facilitators are factors that increase the likelihood of CTA adoption, while barriers are factors that decrease the likelihood of CTA adoption. Table 3 shows the respective factors for each of the 10 categories elicited from the 13 included articles. Privacy and trust turned out to be the most frequent category, while persuasive design and ethical concerns the least frequent category.

**Table 2:** Definitions of the ten categories of factors extracted from reviewed articles.

**S/N**	**Categories**	**Definition/description**
1	Privacy and trust	User's beliefs and concerns about the privacy and trustworthiness of CTAs.
2	Perceived utility	The degree to which a user believes that using a CTA will benefit their and/or public health.
3	Facilitating conditions	Technical affordances, information, and features that facilitate the use of CTAs.
4	Perceived technology threats	User's perceived threats and risks of new technology.
5	Perceived health threats	User's perceived threats and risks of COVID-19.
6	Social cognitive factors	Perceived self-efficacy, social norms, personal attitudes and beliefs about CTAs and social distancing behaviors.
7	Socio-demographic factors	User characteristics, including demographic factors, that influence the adoption of CTAs.
8	Persuasive design	Persuasive features used to motivate CTA adoption and usage.
9	Technology familiarity	User's familiarity with CTAs (e.g., due to compatibility with similar apps used in the past) which fosters self-efficacy and readiness to use them.
10	Ethical concerns	User's concerns about voluntariness, accessibility, affordability, data access, and legal issues associated with CTAs.

**Table 3:** Summary of the factors of CTA adoption.

**Factor**	**Facilitator**	**Barrier**	**#AF**	**#AB**	**#AFB**	**%AFB**
**Privacy and trust (#Article** **=** **13)**						**100%**
Privacy Concern		(27)(23)(15)(16)(22)(20)(26)(17)(29)(18)(21)	0	11	11	92%
Perceived (dis)trust	(27)(16)(23)(24)	(20)(26)	4	2	6	46%
Privacy design/protection	(20)(25)		2	0	2	8%
User-controlled data sharing	(20)		1	0	1	8%
**Perceived utility (#Article** **=** **8)**						**62%**
Perceived usefulness/benefit	(15)(22)(29)(24)		4	0	4	46%
Social benefit	(27)(21)(25)		3	0	3	15%
Personal benefit	(27) (25)	(25)	2	0	2	15%
Personal and social benefit		(25)				
Perceived unnecessariness		(26)	0	1	1	8%
Perceived ineffectiveness		(26)	0	1	1	8%
**Facilitating conditions (#Articles** **=** **7)**						**54%**
Information about app	(22)(26)		2	0	2	15%
Technical concern		(22)(26)	0	2	2	15%
Perceived compatibility	(15)		1	0	1	8%
Innovativeness	(15)		1	0	1	8%
Cues to action	(29)		1	0	1	8%
Perceived ease of use	(24)		1	0	1	8%
Convenience design	(25)		1	0	1	8%
Perceived low adoption rate		(20)	0	1	1	8%
**Social cognitive factors (#Articles** **=** **7)**						**54%**
Attitude towards CTA	(27)(24)(18)		3	0	3	23%
Subjective norm	(27)(15)					15%
SD self-efficacy	(23)					8%
SD response efficacy	(23)					8%
SD response cost	(23)					8%
Perceived trust in others' SDB		(23)	0	1	1	8%
Perceived social safety		(20)	0	1	1	8%
Prosocialness	(21)		0	1	1	8%
**Perceived technological threats (#Articles** **=** **6)**						**46%**
Data security risk		(20)(21)	0	2	2	15%
Perceived susceptibility		(27)(24)	0	2	2	15%
Perceived vulnerability		(16)(23)	0	2	2	15%
Perceived severity		(23)	0	1	1	8%
**Socio-demographic factors (#Articles** **=** **6)**						**46%**
Age	(26)(18)	(20)(21)	2	2	4	31%
Income	(21)	(22)	1	1	2	15%
Living Area	(22)(21)		2	0	2	8%
Gender	(21)		1	0	1	8%
Ethnicity	(21)		1	0	1	8%
Culture	(27)		1	0	1	8%
Work Type		(21)	0	1	1	8%
Public transit frequency	(21)		1	0	1	8%
Health condition	(20)		1	0	1	8%
Education	(20)		1	0	1	8%
**Technology familiarity (#Articles** **=** **5)**						**39%**
IT self-efficacy	(25)(29)		2	0	2	15%
Perceived compatibility	(15)		1	0	1	8%
Privacy self-efficacy	(27)		1	0	1	8%
Technology readiness	(21)		1	0	1	8%
**Perceived health threats (#Articles** **=** **4)**						**31%**
Infection anxiety	(25)(18)		2	0	2	15%
Perceived COVID-19 risk	(24)(21)		2	0	2	15%
**Persuasive design (#Articles** **=** **3)**						**23%**
Tangible reward	(20)(17)		2	0	2	15%
Non-tangible reward	(20)		1	0	1	8%
Location monitoring	(17)(21)		2	0	2	15%
Self-monitoring	(17)		1	0	1	8%
Contact location storage	(21)		1	0	1	8%
Contact location upload	(21)		1	0	1	8%
**Ethical concerns (#Articles** **=** **3)**						**23%**
Voluntariness	(16)(22)		2	0	2	15%
Affordability		(22)(20)	0	2	2	15%
Accessibility		(22)	0	1	1	8%
Data accessor		(22)	0	1	1	8%
Legal issues		(22)	0	1	1	8%

*Work Type: non-essential (0) vs. essential (1), Living Area: non-urban (0) vs. urban (1), Ethnicity: White (0) vs. Hispanic (1), Gender: female (0) vs. male (1), Culture: individualism (0) vs. collectivism (1)*.

*#AF: Number of articles that reported factor as a facilitator, #AB: Number of articles that reported factor as a barrier, #AFB: Number of articles that reported factor as a facilitator/barrier, SD: Social Distancing, SDB: Social Distancing Behavior, IT: Information Technology*.

### Motivational Strategies

The second set of results addresses the second research question, “*What motivational strategies are being implemented to increase the adoption of contact tracing apps*?” The result can be found in the persuasive design category of the factors table (Table 3). The motivational strategies (aka persuasive strategies), which are facilitators of CTA adoption, include tangible reward, non-tangible reward, self-monitoring, social-location monitoring, contact location storage, and location upload.

### Adoption Rates

The third set of results addresses the third research question, “*What are the adoption rates of contact tracing apps among their target audiences?*” Table 4 shows the percentage of participants in each of the 13 studies that were willing to adopt CTAs to curb the spread of the COVID-19 virus. It also shows the percentage of participants in each study that had already adopted (or were already using) CTAs. Eight of the 13 studies investigated and reported this metric (willingness to download, install or use a CTA). The percentage of participants willing to adopt a CTA in the future ranges from 30 to 75%; while the percentage of actual CTA adopters ranges from 37 to 50%.

**Table 4:** CTA adoption rate in each study.

			**Adopters (%)**	
**Author**	**Country**	**Target construct**	**Potential**	**Actual**
Sharma et al. (27)	Public	Intention to install	-	-
Walrave et al. (15)	Belgium	Intention to use	49%	-
Altmann et al. (16)	France, Italy, Germany, UK, US	Intention to install	75%	-
Abuh-ammad et al. (22)	Jordan	Intention to use	72%*	38%*
Kaspar (23)	Germany	Intention to use	-	50%
Jonker et al. (20)	Netherlands	Intention to use	59-66%	-
Thomas et al. (26)	Australia	Intention to download	19%	37%
Cruz et al. (17)	Brazil	Intention to use	-	-
Trang et al. (25)	Germany	Intention to install	-	-
Walrave et al. (29)	Belgium	Intention to use	49%	-
Velicia-Martin et al. (24)	Public	Intention to use	-	-
Jansen-Kosterink et al. (18)	Netherlands	Intention to use	41%	-
Li et al. (21)	United States	Intention to install	59%	-

*Potential: the percentage of participants who intended to download or use CTAs, Actual: the percentage of participants who were using CTAs*.

*: The potential and actual percentage of adopters are not mutually exclusive.

*“-”: Not reported*.

## Discussion

In this review, we synthesized the key findings of the reviewed articles in an overarching fishbone diagram as shown in Supplementary Figure 1. The fishbone diagram allows the target audience of the systematic review to quickly and easily visualize the key factors (organized into ten categories) that influence CTA adoption in one fell swoop. The ten categories include privacy and trust, app utility, usability, facilitating conditions, social-cognitive influence, ethical concerns, perceived technology threats, perceived health threats, persuasive design, and demographic factors. Factors in the privacy and trust category (e.g., privacy concern), followed by factors in the app utility category (e.g., perceived benefit). Most of the factors in both categories [such as privacy concern, perceived trust, and perceived benefit (aka perceived usefulness)] are commonly investigated and found to be significant determinants in the unified theory of acceptance and use of technology (UTAUT) (30, 31). In a nutshell, the fishbone diagram serves as a unified framework for presenting the key factors of CTA adoption from different articles to COVID-19 stakeholders, including the public health authorities, researchers, designers, governments, and policy makers. We hope the findings and takeaways of the framework and the entire review, in the future, will help decision makers and designers create better and more effective CTAs that have the potential of increasing adoption among their target audience. In the next subsections, we discuss the main findings, taking each research question at a time.

### Factors of Technology Adoption

**Figure 3:**
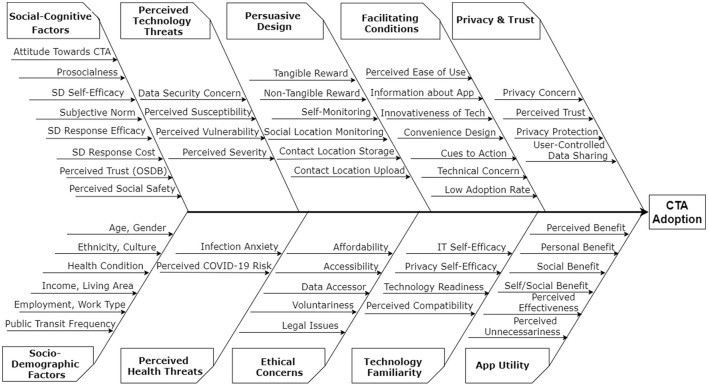
Fishbone diagram showing 56 factors that influence CTA adoption.

Regarding the first research question, we uncovered 10 categories of factors of CTA adoption from the 13 articles included in the systematic review. All 10 categories of factors (comprising facilitators and barriers) are summarized in the fishbone diagram shown in Figure 3. In this section, we discuss the findings and their implications for CTA design, taking each of the categories at a time.

### Privacy and Trust

Privacy and trust turned out to be the most frequent category of factors of CTA adoption, with all 13 articles reporting privacy concern as a barrier and perceived trust, privacy protection, and user-controlled data sharing as facilitators. In particular, privacy concern turned out to be the most frequent factor among the 56 factors elicited, with 11 articles finding it to be a barrier to CTA adoption. Next, perceived (dis)trust turned out to be the second most frequent factor under the privacy and trust category. Four articles (16, 23, 24, 27) found perceived trust to be a facilitator, and two articles (20, 26) found perceived distrust [particularly, in government (26)] to be a barrier. For example, in Thomas et al. 's (26) study, about 11% of the respondents refused to download COVIDSafe due to distrust in the Australian government and the security of the app, with some of the participants believing it “*was not safe and that it could be hacked, resulting in their information being used without their authority*” (p. 2).

Prior research in non-contact-tracing domain shows there is a significant relationship between users' privacy concern and trust in human-computer-interaction (HCI) systems. For example, in social networking (SN), studies showed that the higher the trust users have in SN systems, the lower their privacy concern (32) and vice versa (33). Similarly, in Sharma et al.'s (27) SEM, they found that the higher users' trust in the effectiveness of privacy policy is, the lower their privacy concern, which in turn has a positive direct and indirect effect on users' attitude toward CTAs and intention to adopt them, respectively. Hence, Sharma et al. 's (27) recommended that CTA's privacy statements need to be transparent and informative. They advised CTA developers and sponsors to specify exactly what data is to be collected, what it will be used for, when and how it will be accessed. Moreover, in addition to informing potential users about the data needed by CTAs, Walrave et al. (15) recommended that CTA sponsors should minimize data collection to increase uptake. They also recommended that the amount of time required to read and evaluate privacy terms be reduced through the use a visual presentation to improve comprehension.

The main takeaway from the findings is that if stakeholders can address the privacy and trust related issues, CTA adoption is bound to increase. For example, in Jonkers et al. 's (20) study, the authors recommended that governments implement privacy-preserving CTAs with sufficient realistic features, for example, users should be given control over their data, including the power to decide whom to share their COVID-19 exposure notifications and diagnoses with (20). According to the authors, if security and privacy measures are implemented, this has the potential to produce an adoption rate as high as 64% in the Netherlands.

Furthermore, regarding trust, Altmann et al. (16) recommended that governments should consider delegating the responsibility of digital contact tracing to credible and transparent public health authorities over whom they have little to no control to increase public trust in CTAs. For example, in a choice-based study in the United Kingdom, Wiertz et al. (34) found that CTA adoption rates significantly increased if the National Health Service (rather than the UK government) had ownership and oversight of the CTA. Finally, to foster trust and transparency, experts have recommended that the source code of CTAs be made available to the public. As stated by the Singaporean Foreign Minister, “*We believe that making our code available to the world will enhance trust and collaboration in dealing with a global threat that does not respect boundaries, political systems or economies*” (35).

### Perceived Utility

Utility turned out to be the second most frequent category of factors that influence CTA adoption, with 62% of the articles (8/13) finding empirical evidence on its impact on adoption such as intention to download, install, or use CTAs. Perceived benefits tuned out to be the most recurrent utility-related factor, with seven articles reporting constructs such as perceived benefit (15, 22, 24, 25, 29), personal benefit (25, 27), and social benefit (21, 25, 27) as motivations for CTA adoption. On the other hand, one article (26) found that perceived ineffectiveness and perceived unnecessariness are barriers to CTA adoption. The main takeaway of the utility-based finding is that if users perceived CTAs to be beneficial to them personally and/or socially, they are more likely to adopt them than otherwise. This finding underscores the need for CTA stakeholders and promoters to stress the benefits of CTAs (especially the social benefits) in their sensitization campaigns to improve their adoption (15). For example, Sharma et al. (27) found that individuals were willing to share their personal information on CTAs due to the benefit to the wider community. Specifically, Trang et al. (25) found and recommended that, among undecided citizens, emphasizing social benefits (in addition to privacy design) is more likely to be effective than self-benefits or a combination of self- and social-benefit appeal. Moreover, Li et al. (21) found among United States participants that the perceived benefits of CTAs is a more important determinant of the intention to install them than the perceived security and privacy risks. They reported that users were more willing to install CTAs that collect location data than those that do not due to the additional benefits they provide, including provision of hotspot analysis and information. Finally, Abuh-ammad et al. (22) found that 74.6% of their study's participants believed that CTAs could reduce the rate of COVID-19 infection, 68.8% believed it provides accordable data, 67.8% believed it will reach disadvantaged people, and 48.8% believed it would have more positive effect than other non-digital contact-tracing methods.

### Facilitating Conditions

Facilitating conditions turned out to be the third most frequent category of CTA adoption, with seven of the 13 articles (54%) reporting one or more of the seven constituent factors as facilitators or barriers. The facilitators include cues to action, information about app, perceived ease of use, convenience design, and innovativeness; and the barriers entail perceived low adoption rate and technical concern. Walrave et al. (29) found that cues to action from traditional media (e.g., newspaper, TV/radio station, or magazine) and social media (such as Facebook, Twitter, Instagram, WhatsApp and YouTube) can lead or prompt people to install and use CTAs. This finding, in the light of the emergence of vaccine-resistant COVID-19 variants and their endemic potential (36, 37), stresses the need to advertise CTAs on different media (traditional and social) to increase their uptake. Particularly, Abuhammad et al. (22) emphasized the need for key information about CTAs (including their objectives, description, how they work, who the sponsors are, the potential risks and benefits, and the voluntariness of use) to be provided, as this has the potential of increasing adoption. Moreover, Trang et al. (25) found that convenience design features (such as automatic daily update of app, app running in the background without user intervention, and optimized battery power consumption) can facilitate CTA adoption. Similarly, Velicia-Martin et al. (24) found perceived ease of use to be a facilitator of CTA adoption as well. In the authors' technology acceptance model, perceived ease of use indirectly influences users' intention to use CTAs through perceived usefulness and attitude toward using CTAs. Furthermore, Walrave et al. (15) found that innovativeness (the tendency to be one of the first to adopt a new technology) increases respondents' intention to use CTAs. On the other hand, Abuhammad et al. (22) and Thomas et al. (26) found technical concern to be a barrier to CTA adoption. Particularly, Thomas et al. (26) reported that 24.1% of their respondents cited old phones, limited data consumption and storage space as key barriers to installing CTAs. These barriers can be reduced by ensuring the data size of CTAs is minimized as much as technically possible so that as many users as practically possible can easily download and install them on their smartphones regardless of their phone type or storage space. Another factor that we found to be a barrier to CTA adoption is perceived low adoption rate (20). This barrier can be likened to the small samples hypothesis, which states that decision makers tend to select the options that result in the best payoff in a small sample (38). Specifically, the hypothesis states that people choose to not behave responsibly (e.g., not to adopt and use CTAs) because, regardless of the choices others make (whether they adopt the app or not), it is better most of the time for them (e.g., not having to be concerned with perceived government tracking, privacy issues, battery drainage, app installation space, ineffectiveness of the app due to low adoption, etc.). For this reason, reliance on small samples hypothesis suggests that CTAs such as exposure notification apps may have very little impact on curbing the spread of the virus due to perceived low adoption and usage rates, caused by many people believing that only very few persons in the larger population will adopt or have adopted the app. One plausible explanation for most people not wanting to adopt CTAs due to perceived low adoption is shared guilt (39). People assume that even if they decided to act responsibly (i.e., adopt and use a CTA), others would decide to behave recklessly (not to adopt and use the app). Hence, the individual's choice to not act responsibly and the guilt that comes with it are attributed to others by the individual. To address this challenge, Plonsky et al. (40) proposed a gentle rule enforcement and the implementation of utilitarian features that increase the personal benefits users derive from installing and using a CTA, which can help users meet the gentle rule easily. The gentle rule can help to save time and minimize efforts. One of such features is the ability to use one's CTA to book a vaccination appointment, download one's vaccination certificate, and use it to access public facilities without having to fill long forms, go through long checks, or be physically screened or tested for COVID-19 (40). The enforcement of gentle rules (such as the display of a green signal on one app's screen in order to enter stores and public facilities or to travel) helped break the chains of COVID-19 transmission in China (41).

### Social Cognitive Factors

Social-cognitive factors turned out to be the fourth most frequent category of factors that influence CTA adoption, with 54% of the articles (7/13) finding empirical evidence supporting its likelihood to impact users' intentions to adopt CTAs. The factors include prosocialness, subjective norm, perceived trust in others' SDB, SD self-efficacy, SD response efficacy, SD response cost, and perceived social safety. All of these factors, except for perceived trust in others' SDB and perceived social safety, are facilitators. Li et al. found that individual characteristics such as prosocialness [operationalized as selflessness, empathy, and altruism (42)] had a positive impact on the intention to use CTAs. In other words, the more people care for the welfare of others (social and health), the more likely they are to use CTAs to fast-track the curbing of the spread of the COVID-19. Similarly, Sharma et al. (27) and Walrave et al. (15) found that subjective norms [such as the belief that friends and/or social influencers expect one to install a CTA (27)] positively influence the intention to use CTAs.

Furthermore, Kaspar (23) found that three factors related to social distancing—including belief in one's ability to social distance (SD self-efficacy), the effectiveness of social distancing (SD response efficacy), and the exhausting nature of social distancing (SD response cost)—positively influence the motivation to use CTAs. These findings are based on the assumption that CTAs have the capability of informing users about the need to social distance or prompting users to social distance in public spaces. In this regard, one would have expected that the perceived cost of social distancing would have negatively influenced the motivation to use CTAs as the author (Kaspar) hypothesized (23). However, it turned out that the higher the study subjects find it exhausting to social distance, the higher their motivation to use CTAs. One plausible explanation for this finding is the benefit social distancing behaviors foster (reduction of the likelihood of being infected by COVID-19). However, further studies are needed to confirm this finding, especially among other populations than the German population associated with the finding.

On the other hand, Kaspar (23) found that perceived trust in others' SD behavior negatively influences the motivation to use CTAs. This finding suggests that the higher a person's belief in other people “*trying their best to avoid getting too close to other people in public life*” (23), the less likely they are to use CTAs. This finding suggests that the more people believe in the effectiveness of physical distancing, the less likely they are to deem CTAs useful. With that said, given that the relationship between this social-trust related factor and motivation to use CTAs is weak in Kaspar's (23) study, further studies are required to confirm the finding.

Finally, Jonker et al. (20) found that the more people feel safe in large groups, the less likely they are to adopt CTAs. According to Jonker et al. (20), this is due to the fact that such people considered the chance of getting infected with COVID-19 and being seriously ill if infected to be small. Consistent and continuous campaign on traditional and social media may help in increasing public awareness about the transmissibility of COVID-19 if preventive measures such as social distancing and wearing masks are not taken seriously and the potential of the virus causing death. Moreover, the endemic potential of COVID-19 and the emergence of new variants (such as the Delta strain), which have the potential of causing breakthrough infections among vaccinated people, should be emphasized as well (36).

### Socio-Demographic Factors

Socio-demographic factors turned out to be the fifth most frequent category of factors that influence CTA adoption, with 46% of the articles (6/13) reporting one or more of the associated factors. Six of the 13 articles reported a total of 10 socio-demographic factors such as income, living area, age, gender, ethnicity, culture, work type, etc., which influence CTA adoption. Li et al. (21) reported seven of the socio-demographic factors. The authors found that higher income people, urban residents, younger people, males, essential workers, Hispanics (compared with white), and commuters with higher transit frequency are more likely to adopt CTAs. It is counterintuitive that older people and essential workers are less likely to adopt CTAs. As we know, older people and essential workers (such as nurses and grocery salespersons) are more likely to be exposed to COVID-19 given that they are more susceptible and have to be out every day rendering essential services to the public, respectively. The finding that older people are less likely to adopt CTAs is inconsistent with Jansen-Kosterink et al. 's (18) and Thomas et al. 's (26) findings. However, it is consistent with Jonker et al. 's (20) finding, in which the authors found that older people (75 and above) are less likely to use CTAs compared with younger people (15–34). To address this unwillingness to use CTAs, Jonker et al. (20) suggested that a tailored communication strategy be used to maximize the uptake of CTAs among older people. The mixed findings with regard to the effect of age on CTA adoption requires further studies to gain more insights into why age negatively or positively influences CTA adoption. Moreover, Li et al. (21) found that people with higher transit frequency are more likely to adopt CTAs. A plausible explanation is that this group of people is more likely to be exposed to COVID-19 given the relatively higher number of times the people have to use public transit systems such as the train and the bus. Moreover, Jonker et al. (20) found that people with higher education, and severe health conditions (e.g., lung disease, kidney disease, or compromised immune system) are more likely to use CTAs. This may not be surprising as people with higher education are more likely to be well-informed about the COVID-19 virus, and people with severe health conditions are more likely to be susceptible and vulnerable to the virus.

### Perceived Technology Threats

Perceived technology threats turned out to be the sixth most frequent category of factors that influence CTA adoption, with 46% of the articles (6/13) finding empirical evidence on its likelihood to negatively impact users' intentions to adopt CTAs. The most frequent barrier to CTA adoption is data security concern (20, 21), followed by perceived susceptibility (24, 27), perceived vulnerability (16, 23), and perceived severity (23) (see Appendices 1, 2 for the definitions, differentiations, and discussions of these technology-threat-related constructs). In Jonker et al. 's (20) study, 50% of the participants were concerned about the security of the CTAs. Moreover, Altmann et al. 's (16) reported they 35% of the participants feared that using CTAs could make their phone vulnerable to hackers. Similarly, Li et al. (21) reported that participants were concerned about data breach risk (stored data stolen by outside hackers), secondary data use risk (long-stored data used for other purposes), and the re-identification risk of users who tested positive, with the second concern significantly reducing CTA adoption intention. One common approach to reduce the perceived security threats and risks associated with the usage of CTAs is the enactment of user data anonymization/deidentification. This has the potential of increasing adoption of CTAs, especially those that require user data such as location, COVID-19 one-time key, phone number, etc. For example, in Altmann et al. 's (16) study (see Supplementary Table 1), 60% of the participants responded that they would consent to making their deidentified data available to research.

### Technology Familiarity

Technology familiarity turned out to be the seventh most frequent category of CTA adoption, with four articles (69%) reporting factors such as IT self-efficacy, privacy self-efficacy, perceived compatibility, and technology readiness as facilitators. Walrave et al. (15), Trang et al. (25), and Walrave et al. (29) found that perceived compatibility/IT self-efficacy (e.g., having the knowledge and the necessary resource to use CTAs) facilitates users' intentions to install or use CTAs. Particularly, Sharma et al. (27) found that privacy self-efficacy increases CTA adoption intentions. As a way of reducing cognitive workload, Walrave et al. (15) recommended a visual presentation of privacy policy to improve comprehension and minimize the amount of time required to read and evaluate CTA's privacy terms. Finally, Walrave et al. (15) found that technology readiness (predisposition to use a new technology) increases respondents' intention to install/use CTAs.

### Perceived Health Threats

Perceived health threats turned out to be the eighth most frequent category of factors that influence CTA adoption, with 31% of the articles (4/13) reporting two constituent factors [COVID-19 infection anxiety (18, 25) and perceived COVID-19 risk (21, 24)] that facilitate CTA adoption. (see Supplementary File 2 for the definition and clarification of the factors). Li et al. (21) found that the higher the COVID-19 risk perception of their study participants was, the more inclined they were to install and use CTAs. For this reason, the authors recommended that marketing campaigns on CTAs appeal to protecting the users and the public against COVID-19 by using digital contact tracing to augment manual contact tracing. Moreover, Jansen-Kosterink et al. (18) found that infection anxiety (i.e., fear of COVID-19) is a determinant of the acceptance of CTAs. In their study among Dutch adults, the authors reported that 16% of the responders were afraid of contracting COVID-19 and 80.7% of them were neutral. Other perceived health threat factors that were investigated included perceived susceptibility (29), perceived vulnerability (23), and perceived severity (23, 29). However, these factors did not significantly influence CTA adoption. Hence, further work needs to be done to confirm or refute these preliminary findings.

### Persuasive Design

Perceived technology threats turned out to be the seventh most frequent category of factors that influence CTA adoption, with 23% of the articles (3/13) finding empirical evidence on its likelihood to impact users' intentions to adopt CTAs. This category of factors addresses the second research question. Four of the 13 articles found factors related to persuasive design as drivers of CTA adoption. The factors include tangible reward, self-monitoring, social-location monitoring, contact location storage (on device), and contact location upload (to server). For example, Cruz et al. (17) found that five of these factors (self-monitoring, social-location monitoring, contact location storage, location upload, and reward) have the potential to motivate CTA adoption. Self-monitoring has to do with the tracking the number of contacts (e.g., infected persons) a user has come in close contact with, within a given period. On the other hand, social-location monitoring is the tracking of infection-rate information about a given location, e.g., the number of infected persons that currently reside in this location or passed it over a given period. This information helps users to make informed decisions about their daily itineraries. Cruz et al. (17) found that over 50% of their study participants requested the self-monitoring and social location monitoring features. Social-location monitoring can only be made possible if users are willing to track their locations (e.g., locally on their device) and upload them whenever they test positive. Li et al. (21) reported that, in their study, users were more willing to use CTAs that support the local storage of their locations on their device and their upload when they tested positive so that alerted (exposed) users could recall their recent whereabout where the infection might have occurred. Moreover, the uploaded location information would help healthcare workers analyze COVID-19 infection hotspots and provide the public with useful information that could help them make useful decisions about their itineraries. Finally, regarding reward, Cruz et al. (17) found that more study participants were more willing to share their locations when they were offered access to the health system (76%) and/or a tangible reward (71%) than when they were offered a non-tangible reward (65%). Similarly, Jonker et al. (20) found that financial reward as well as non-financial reward (e.g., permission to socialize) encourages users to use CTAs. Specifically, they found that respondents preferred CTAs that offer them additional benefits such as a small monetary reward of €5 (US$ 6) or €10 (US$ 12) per month, permission to gather in small groups and free COVID-19 testing after receiving an exposure alert. These reward-based findings are in tandem with that of a choice-based study in which Wiertz at al. (34) found that CTA adoption rates significantly increased if app use was linked to priority testing for COVID-19 when alerted CTA users were in self-isolation.

### Ethical Concerns

Ethical concerns turned out to be one of the two least frequent categories of factors that influence CTA adoption, with 23% of the articles (3/13) reporting affordability (20, 22), accessibility (22), data accessor (22) and legal issues (22) as barriers, and voluntariness (16, 22) as a facilitator. Data accessor entails who gets the mandate to access CTA data, while legal issues border on questions such as “*could users still consent if they are legally mandated to install a CTA?*” (22). Abuhammad et al. (22) reported all five factors should be taken into consideration when deploying a CTA. Regarding data access, for example, 85.6 and 82.2% of the respondents agreed that the World Health Organization and contact tracing software companies, respectively, should have access to the collected data. However, 79% of the respondents disagreed that people who used the CTA should be allowed to control or manage it. This is an indication that this group of participants (Jordanians) are more in favor of the health and tech authorities (e.g., World Health Organization) controlling and managing CTAs in order to curb the spread of COVID-19. This finding is consistent with the finding among participants in the United Kingdom, who preferred the National Health Service to the UK government regarding having ownership and oversight of CTAs (34). One plausible explanation for the preference of health institutions and tech companies is that respondents believed that they are more trustworthy and/or competent to handle and manage COVID-19 contact tracing compared with the government. Regarding voluntariness, Altmann et al. (16) investigated two regimes of CTA installation: opt-in and opt-out. The opt-in is voluntary installation by user, and the opt-out is automatic installation by mobile phone providers (government proxy). They found that 74.8% of the respondents in the opt-in group would probably or definitely download the CTA if it was available on the app store, compared with 67.7% of the respondents in the opt-out group, who said they would probably or definitely keep the app on their phone if installed automatically. This is an indication that the opt-in regime is more likely to be effective. Finally, affordability may be a barrier to CTA adoption. Experts have expressed concern about the legal and ethical implications of making CTAs compulsory, especially among those people who do not have access to the Internet or a smartphone (22). For example, Jonker et al. (20) found that the non-ownership of phone and having to spend money out of pocket to own a CTA may hamper the adoption of CTAs. Hence, in order for wide adoption of CTAs, COVID-19 stakeholders have advocated fostering ethics including equitable access in the design of CTAs to ensure that they reach a critical mass of the population (43). For example, governments can provide low-cost devices that support contact tracing to individuals that do not have Bluetooth-enabled smartphones (44).

### CTA Adoption Rates

Regarding the third research question, “*What are the adoption rates of CTAs among their target audiences*?,” we elicited three adoption variables: intention to download, intention to install, and intention to use (see Table 4). Regarding intention to download CTAs, we found one study that reported the adoption rate among potential and actual users. Among Australian study participants, Thomas et al. (26) found that 37% of them had adopted the COVIDSafe app, and 19% intended to download the app. However, 28% refused to download the app, and 16% were undecided. The authors reported privacy and technical concerns as the main reasons why these user groups did not intend to download the app or were undecided. Similarly, regarding intention to install CTAs, we found one study that reported the adoption rate among potential users. Li et al. (21) found in their study that 59% of American participants were at least somewhat willing to install the app. It is noteworthy that, in the same study, the authors found 76% of the participants were at least somewhat willing to report their COVID-19 diagnosis if they tested positive. It is interesting to know that there were more people willing to report their diagnosis (76%) than those willing to download the app (59%). A plausible explanation for the numerical difference (17%), i.e., those that were willing to report their diagnosis, but not willing to download the app, include privacy and data security concerns, which the authors reported as the main barriers to CTA adoption.

Moreover, regarding intention to use CTAs, five studies (15, 18, 20, 22, 29) reported an adoption rate ranging from 41 to 75% for potential users and 37 to 50% for actual users. On one hand, Jansen-Kosterink et al. (18) and Walrave et al. (15, 29) found that 41% of Dutch participants and 49% of Belgian participants, respectively, were willing to use CTAs to curb the spread of the COVID-19 virus in the future. Jansen-Kosterink et al. (18) reported coronavirus anxiety and positive attitude toward technology as the facilitators of CTA adoption among the Dutch participants. Moreover, Walrave et al. (15, 29) reported that perceived usefulness/benefit (strongest factor), perceived compatibility, subjective norm, and innovativeness can facilitate the adoption of CTAs among Belgian participants. In both studies, privacy concern was reported as a barrier, which might have been the main reason why over 50% of participants in each of the study did not agree to download CTAs in the future. On the other hand, Abuh-ammad et al. (22) and Altmann et al. (16) found that 72% of Jordanian participants and 75% of French, Italian, German, British, and American participants were willing to use CTAs to contain the spread of the virus in the future. In the latter study, compared with French, Italian, and British participants, German and American participants were less willing to adopt CTAs. With that said, in Abuh-ammad et al. 's (22) study, perceived benefit and voluntariness were the key reasons why the target participants wanted to use CTAs in the future. Similarly, in Altmann et al. 's (16) study, voluntariness as well as automatic installation (particularly, that supports an opt-out regime) by mobile phone providers tend to increase users' intention to use CTAs. However, Altmann et al. 's (16) reported privacy concern, cybersecurity concern, and lack of trust in government as the primary barriers to CTA adoption among the French, Italian, and British participants, German and American participants. Similarly, Abuh-ammad et al. 's (22) study reported privacy concerns, ethical concerns (e.g., accessibility, technical problem, legal problem, and participation cost) and limited information as possible barriers to the use of CTAs among Jordanian participants. To increase CTA adoption, regardless of country, the elicited barriers (particularly privacy concern) must be addressed.

Finally, we found that there is a stark contrast between two studies with regard to the potential and actual adoption rates (Table 4). Particularly, in Abuh-ammad et al. 's (22) study, the potential adoption rate (72%) is roughly double the actual adoption rate (38%). However, the reverse is the case in Thomas et al. 's (26) study: the actual adoption rate (37%) is approximately double the potential adoption rate (19%). Although both groups of authors did not provide an explanation for the difference, Abuh-ammad et al. 's (22) did recommend the need to motivate the target population to download the CTA, for example, by using social media and governmental channels. Specifically, in the second study, Thomas et al. 's (26) explained that their finding (37% adoption rate) is in accordance with an Australian study (45), in which 44% of the participants reported downloading the COVIDSafe app. However, they added, compared with other similar studies, the potential adoption rate of 19% in their study was lower. The authors cited an example study conducted in Ireland, in which 58% of the participants said they would download a CTA that was not yet available, and 25% said they probably would. They also cited Altmann et al. 's (16) study, included in this review (see Table 4), in which 75% of the participants said they definitely or probably would install a CTA. In this regard, future studies can investigate, in a longitudinal study, the likelihood of participants following through with their intentions to download, install, and/or use a CTA.

### Recommendations

Having presented the key factors of CTA adoption based on 13 included articles, we provide design recommendations to improve future iterations of CTAs and increase adoption. The recommendations are based on the first two research questions as well as on the most frequent factors of CTA adoption.

#### Recommendation 1: Implementing and Communicating Privacy Protection Measures

Privacy concern turned out to be the most frequent factor. To increase CTA adoption through privacy protection, we recommend the following design guidelines based on the findings from the privacy-related articles.

1. Minimize privacy concern by reducing the amount of personal identifiable information collected (46).
2. Allow users to decide or know what data will be collected, what they will be used for, and who will be allowed to access them, the purpose for which they will be used, and for how long the data will be stored. Hence, collected data should only be retained for as long as is necessary to serve the specific purpose for which it was collected (47).
3. Give users choices when it comes to the amount of data they are willing to provide or share, with each choice having its pros and cons. For example, users can be given the choice of providing their location data with the additional benefit of accessing hotspot information and analysis (21).
4. Implement visual presentation of privacy policy to improve comprehension and reduce the amount of time required by the user to read and evaluate the terms (15).

It is noteworthy that while we recommend the need for CTAs to foster privacy based on the review's findings (privacy concern being the most frequent factor of adoption), we acknowledge that there is a need to strike a balance between privacy and effectiveness, as focusing on either goal alone may result in one suffering. As often said, there is a cost for every action. For example, increasing privacy [e.g., non-collection of location data (47)] can substantially reduce the effectiveness of CTAs, which in turn can lead to undesired outcomes, including the impairment of public health and social welfare. In general, in healthcare service delivery, the ease of personalized information retrieval can only be made possible by the collection of personal data and the possibility for the user to control their health status without having to depend on the availability of healthcare professionals. This is somewhat the case when it comes to digital contact tracing apps. For example, in symptom checkers, for artificial-intelligence enabled chatbots to provide personalized services to the user, there may be a need for the user to provide some personal information about their health (48). While we acknowledge that the need to strike a balance between privacy and effectiveness is critical to the success of CTAs, finding the right balance can be challenging as the concept of privacy can be highly context-specific, culture-specific, country-specific, and even individual-specific (49).

#### Recommendation 2: Emphasizing CTA Utility and Improving It by Persuasive Design

Eight articles (15, 21, 22, 24–27, 29) reported factors associated with utility, such as usefulness, benefit, and effectiveness, which have the potential to increase CTA adoption. Hence, we recommend that CTA sponsors emphasize the benefit (especially the social) to public health, e.g., in traditional media, online websites, and social media. For non-users, the focus should be on the need to install and use the app to curb the spread of the COVID-19 virus (especially to most vulnerable groups of people such as the elderly) through getting notified of a possible exposure, having to self-quarantine for 14 days, and testing for COVID-19 if symptoms are noticed. In other words, using CTAs for the “greater good” of society should be emphasized in marketing campaigns (21). Trang et al. (25) demonstrated that emphasizing the societal benefits of CTAs is more likely to lead to a higher willingness to adopt CTAs compared with emphasizing the personal benefits. Moreover, Li et al. (21) showed that prosocialness has a positive impact on CTA adoption.

Apart from the basic COVID-19 exposure notification, participants asked for more useful features which border on persuasive design. One of the persuasive features they requested for is self-monitoring: the ability to track the number of contacts and the location of their exposure (17). Recent research by Oyibo and Morita (50) demonstrated that a CTA equipped with self-monitoring (tracking of user's daily contacts and exposure time) is more likely to be adopted than the control version unequipped with self-monitoring. Secondly, participants requested social (location) monitoring: the ability to monitor the number of COVID-19 infections in a given locality or commercial center (17, 21). To ensure that the monitoring features can be implemented in CTAs, some of the participants were willing to store their location in their device for a given period of time and upload it to a central server in the event that they tested positive (21). Finally, to promote and encourage the adoption of CTAs, tangible reward (incentives) can be offered to their users. This can be achieved through the conversion of virtual reward (which the user accrues through the use of the app and data provided) to tangible reward. In the event that COVID-19 becomes endemic [which some health experts have predicted (51)], provision of tangible reward (such as free testing for COVID-19 if alerted by the app, government-subsidized treatment for COVID-19 patients, etc.) may be necessary to sustain the use of CTAs going forward (17, 20). Provision of this kind of tangible reward can be rationalized thus: by using a given national CTAs regularly, users are contributing to the reducing the spread of the COVID-19 virus, which reduces the number of infected/hospitalized patients and the attendant healthcare cost. Hence, some of the saved healthcare cost can be channeled into supporting people who are contributing to minimizing the spread of the virus by using the government-approved CTA.

#### Recommendation 3: Fostering Public Trust Through Delegation and Transparency

Apart from privacy concern, research shows that users have less trust in governments and technology companies compared with public health authorities (52). Particularly, some users believed that digital contact tracing was a government surveillance scheme, which might be used “adversely” against them now or in the future (i.e., when the pandemic is over). Hence, they are unwilling to participate, especially when CTAs are collecting or tracking personal data such as location, contacts, etc. Six articles reported perceived trust (16, 23, 24, 27) and perceived distrust (20, 26) as facilitators and barriers, respectively, to CTA adoption. To reduce the distrust in CTAs, experts recommend that reputable and credible public health authorities [e.g., Health Canada, National Health Service (34), etc.] rather than governments should be allowed to take ownership of digital contact tracing, with little or no oversight from the government, just as is the case with manual contact tracing (16). This has the potential of increasing trust in CTAs and improving adoption. Another unique step to improve trust and adoption is the fostering of transparency, for example, through the making of CTA source code available to be public (35, 48).

#### Recommendation 4: Implementing and Communicating Data Security Measures

Five articles reported factors related to data security as barriers to CTA adoption. The factors include data security risk (20, 21), perceived susceptibility to security breach (24, 27), perceived vulnerability to data hacking (16, 23), and perceived severity of cybersecurity threats (23). Hence, we recommend that CTA sponsors should put measures in place to ensure user data are securely protected. Particularly, they should inform users about the measures that have been put in place to ensure that their data is safe and secure. Such measures can include storage of user data in their local device (e.g., location, randomly generated contact identifications), anonymization and deidentification of user data, storage of user data on a server for only the period within which it is needed, among others (21).

#### Recommendation 5: Fostering Compatibility and Consistency in App Design

Five articles reported factors related to familiarity with technology, all of which are facilitators of CTA adoption. The facilitators include perceived compatibility (with existing similar apps) (15), IT self-efficacy (which increases with perceived compatibility) (25, 29), privacy self-efficacy (27), and technology readiness (21). These facilitators indicate that the more the design of CTAs is similar to the apps users have used before, the more likely they are to adopt them due to familiarity and perceived self-efficacy. Hence, we recommend that CTAs, as much as possible, be compatible and consistent with de facto design standards employed in most apps to reduce the learning curve and increase ease of use.

### Research Opportunities

The reviewed studies have some gaps and limitations, which offer opportunities for future research on CTAs. First, as shown in Figure 2, most of the studies were conducted among European populations (66.7%) and North American populations (13.3%). The findings in Western countries may not generalize to non-Western countries such as Africa, Asia, and South America. This calls for more research (especially cross-cultural comparative studies). This may help us to uncover how the existing findings generalize to the understudied continents and how different countries/cultures in different continents (e.g., Canada vs. Nigeria) differ in their perceptions of and attitudes toward CTAs. Secondly, we found that most of the studies were description-based (69.2%) and conducted in the first half of 2020 (69.2%) when the public was yet to know much about CTAs and there were a lot of misconceptions and mis-speculations about CTAs. Over the months, some people have come to have a better understanding of CTAs due to the availability of the various deployed apps worldwide and more information about how they function and their utility in curbing the spread of COVID-19. The availability of more information [particularly the endemic potential of the COVID-19 virus (51)] may influence the perception of CTAs and people's willingness to use them moving into the future. Hence, we recommend more studies (especially those focused on already deployed national apps) be carried out in the future to see how the perceptions and attitudes of the public have changed over time given new information about CTAs and COVID-19. Finally, the results of the review show that very little has been done on the potential effectiveness of persuasive design in increasing CTA adoption. We only found three articles (17, 20, 21), which examined the potential effectiveness of persuasive strategies such as reward, self-monitoring, social-location monitoring. Moreover, there are other persuasive strategies [e.g., from the persuasive design system model (53)], which have the potential to motivate CTA adoption as well. Such persuasive strategies include tailoring, social learning, and social comparison (54). We recommend that future work investigate the potential effectiveness of these persuasive strategies, as they hold promising prospects in increasing CTA adoption.

### Strengths and Contributions

Our systematic review has a number of strengths compared with prior reviews. The main strength of our review is that we adopted a comprehensive search strategy (formal and informal) that involved seven databases from health science and computer science. For example, PubMed and CINAHL focus on topics in the health (life science) domains, while Scopus, IEEE Xplore and ACM Digital Library focus on technology- and application-based topics. Hence, in our database search, we were able to uncover as many articles as possible as of the time of working on the review. Another strength of our review is that we focused on peer-reviewed articles only unlike prior reviews that focused on non-peer-reviewed articles as well as gray literature [e.g., (7)]. The third strength of our review is that we were able to focus on more recent articles which prior reviews such as Braithwaite et al. (7) did not focus on due to the authors' relatively early work and publication in the earlier part of the pandemic in 2020.

By this systematic review, we have made a number of contributions to the literature on CTA adoption. The first contribution is that we elicited 56 factors of CTA adoption, presented in a fishbone diagram. The fishbone diagram, first presented in this systematic review, serves as an overarching preliminary framework for presenting the main drivers and barriers to CTA adoption to stakeholders such as researchers, designers, public health authorities, governments, and policymakers. This will enable CTA stakeholders (researchers and non-researchers) to quickly and easily identify the main factors that influence CTA adoption. Particularly, it will enable CTA decision-makers and designers to focus on the key motivational design factors [e.g., persuasive design (54)] necessary to create better and more effective CTAs that have the potential of increasing adoption among non-adopters (10).

The second contribution is that we provided definitions and clarifications of constructs/factors of CTA adoption (see Supplementary File 2) that may be confusing to readers and have been misunderstood in prior literature, respectively. The definitions will help readers understand the review and the presented findings better. Moreover the clarification of the perceived-risk-related constructs such as perceived susceptibility, perceived vulnerability, perceived severity, and perceived likelihood will help researchers communicate in a commonly understood language and operationalize the respective constructs appropriately.

The third contribution is that we provided recommendations and identified unfilled gaps in the current literature (e.g., related to understudied populations, app type, and study type), which researchers can leverage in future design of CTAs and address in future research efforts, respectively (10). For example, our review revealed that there is no research on CTA technology acceptance among the African population, which is an integral part of the COVID-19 pandemic. The review also revealed that there is little to no cross-cultural research on CTA technology acceptance, especially between Hofstede's (55) individualist and collectivist societies.

### Limitations

Despite its contributions, our review has limitations, which can be addressed in future work. The first limitation is that we only focused on COVID-19 CTAs by including the term “COVID-19” and its alternative names in our set of search terms. This tends to limit our findings to COVID-related CTAs and not CTAs in general. Hence, our reviews did not cover CTAs [e.g., Ebola-related CTAs (56)] prior to the COVID-19 pandemic. The second limitation of the study is that not of all of the included studies in the systematic review established an empirical relationship between the presented factors and CTA adoption. For example, in Abuh-ammad et al. (22), the study was not based on quantitative analyses such as correlation analysis, regression analysis, path analysis, or analysis of variance. Rather, it was merely based on counts, for example, the percentage of participants that think a given construct (e.g., voluntary participation, privacy of information, or accessibility) is important to the use of CTAs. Hence, more quantitative research needs to be done regarding the factors in question to validate the findings of previous authors that did not show an empirical relationship between the facilitator/barrier and CTA adoption constructs. The third limitation of our review is that we could and did not control for the various risks of biases that we identified in the review. In general, one way to reduce biases such as studies being carried out at different stages of the pandemic, studies applying different research designs and stimuli, studies having varying demographic make-up of studies, and studies using different methods of data analyses, is to control for these factors. However, given the small number of included studies (*n* = 13) and the main goal of the review was not to carry out a comprehensive comparative analysis between studies, we did not control for these factors, namely, stage of pandemic, research design/stimulus, demographic variable, and data analysis method. Future systematic reviews at a time in which a substantial number of CTA studies have been published can address these limitations, e.g., by carrying out a comprehensive comparative analyses between similar studies segmented by factors such as research design, research stimuli, and data analysis method.

### Future Work

We would like to acknowledge and state that the elicited factors affecting CTA adoption shown in the fishbone diagram are not exhaustive given that there may be studies presenting other factors that we missed in our database search and/or that new studies have been published after our search. For example, in the context of privacy versus effectiveness, such studies may contain choice of wireless technology (Bluetooth Low Energy vs. GPS) as a possible technical factor affecting CTA adoption. For instance, the failures in Bluetooth Low Energy technology (e.g., inaccuracies in converting the signal strength into distances), especially in indoor environments (57), may result in GPS technology being preferred by some CTA users. Another possible factor of CTA adoption that may be investigated in future work is probability of regret. Despite the importance of key factors such as privacy and trust, probability of regret may turn out to be a significant factor in CTA adoption. Probability of regret holds that in decision-making, individuals might anticipate regret and, for this reason, incorporate their desire to eliminate or minimize it in the final decision or choice they make. It will be interesting in future work on CTA adoption to investigate the role probability of regret, visa-vis privacy concerns and other identified factors, plays in CTA adoption (58, 59). For example, are people prepared to tradeoff privacy to avoid regretting not adopting and using CTAs to help curb the spread of the COVID-19 and return the economy to normalcy? (60). A third possible factor of CTA adoption that may be investigated in future work is small frequently experienced costs. A number of CTAs provide utility that goes beyond contact tracing and exposure notification, which may cost those not using them, for example, due to privacy concerns and lack of trust in CTA stakeholders, something. For instance, Aarogya Setu, the Indian CTA, allows vaccinated users to download their vaccination certificate on their smartphone and use it to easily access public places (61). As stated by Plonsky et al. (40), “*while both people with and without the certificate will be able to access public facilities and workplaces, not carrying a certificate will incur a small cost in time and effort*” (p. 23). It will be interesting in future research to investigate how small frequently experienced costs such as taking time and effort to access a public facility, vis-a-vis privacy concerns and limited trust of CTAs, may influence their adoption. While this may not get all people to use CTAs (especially those with high hesitancy), Plonsky et al. (40), predicted that the small frequently experienced costs (e.g., being required to fill certain declaration forms, undergo and pass certain physical tests at the entrances of public facilities) may help increase CTA adoption and vaccine uptake as well. Hence, the authors recommended that policy makers and app designers should make the common experience from using CTAs better than the common experience from not using them (40).

## Conclusions

We have presented a systematic review of the factors of CTA adoption. The review identified 13 relevant articles, from which a total of 56 factors, comprising facilitators and barriers, were extracted. These factors were classified into 10 thematic categories, which are visualized in a fishbone diagram for easy comprehension, discussion, and brainstorming. The 10 thematic categories include privacy and trust, utility, facilitating conditions, perceived technology threats, perceived health threats, social-cognitive factors, ethical concerns, technology familiarity, persuasive design, and socio-demographic factors. Privacy concerns turned out to be the most frequent influencing CTA adoption, followed by perceived benefit, perceived (dis)trust, perceived data security risk, and technology familiarity. The key recommendations based on the most frequent categories of factors include: (1) users' privacy concerns should be alleviated by implementing and communicating privacy protection measures, which include minimization of user data collected and giving users control over their data; (2) Stakeholders should emphasize the utility of CTAs (especially to the elderly) in the context of the “greater good,” and improve it through persuasive design such as self-monitoring and tangible reward; (3) Government should delegate the responsibility of digital contact tracing to public health authorities (with little or no oversight function) and make source code available to the public to foster trust and transparency; (4) Stakeholders should implement and communicate data security measures aimed to protect user data against cyberattacks, hacking and misuse; (5) CTAs should be designed to be consistent and compatible to existing similar apps to reduce the learning curve, increase users' perceived self-efficacy, and perceived ease of use (62). Future studies should focus on non-Western countries such as African, Asian and South American countries which are underrepresented in the current research on CTA adoption. Particularly, they should conduct cross-country/cultural research involving Western (developed) and non-Western (developing) countries to uncover how these groups of CTAs users significantly differ. Findings from cross-country/cultural research will go a long way towards advancing the current body of knowledge that is mainly based on Western countries.

## Data Availability Statement

The original contributions presented in the study are included in the article/Supplementary Material, further inquiries can be directed to the corresponding author.

## Author Contributions

KO, KS, and AO were involved in the retrieval of the articles from the databases and tabulation of the data in a spread sheet. Moreover, KO wrote the article and PM helped with the review. All authors contributed to the article and approved the submitted version.

## Conflict of Interest

The authors declare that the research was conducted in the absence of any commercial or financial relationships that could be construed as a potential conflict of interest.

## Publisher's Note

All claims expressed in this article are solely those of the authors and do not necessarily represent those of their affiliated organizations, or those of the publisher, the editors and the reviewers. Any product that may be evaluated in this article, or claim that may be made by its manufacturer, is not guaranteed or endorsed by the publisher.

## Abbreviations

ACM: Association for Computing Machinery
COVID: coronavirus disease
GAEN: Google/Apple Exposure Notification
PRISMA: Preferred Reporting Items for Systematic Review and Meta-analysis
PRISMA-P: Preferred Reporting Items for Systematic Review and Meta-analysis Protocols
RQ: research question
SD: social distancing
WOS: Web of Science.
