# Supplementary material for: Factors Influencing the Adoption of Contact Tracing Applications: Systematic Review and Recommendations
Source: Front Digit Health. 2022 May 3;4:862466. doi: 10.3389/fdgth.2022.862466 (PMC9110790; doi:10.3389/fdgth.2022.862466)
Supplement: Supplementary file 1 [file Data_Sheet_1.pdf]

Supplementary File 1: Factors Influencing the Adoption of Contact Tracing Applications: Systematic Review and Recommendations

Kiemute Oyibo; Kirti Sundar Sahu; Arlene Oetomo; and Plinio Pelegrini Morita

Supplementary Table 1. Extracted data from included articles. CTA: Contact Tracing App, QT: Quantitative Study DF: Demographic Factor, SEM: Structural Equation Model, UTAUT: Unified Theory of Acceptance and Use of Technology, TAM: Technology Acceptance Model, HBM: Health Belief Model, PMT: Protection Motivation Theory, RM: Regression Model, T: Technology related, H: Health related, "*"Perceived susceptibility was labelled as perceived vulnerability in the original paper.

**Authors**	**Study Date**	**App Type**	**Target Audience**	**Study Type**	**Outcome Variable**	**Facilitators/** ***Barriers***	**Moderator/ DF Effect**	**Findings/Takeaways**	**Recommendations**
Sharma et al. [1]	April-May, 2020	General knowledge of CTAs	Public/ Facebook (n=714, age = 18+)	QT (SEM: Composite model)	Intention to install (R^2=51%)	-Attitude<br>-Subjective norm<br>-Privacy self-efficacy<br>-Personal benefit<br>-Social benefit<br>-Perceived privacy policy effectiveness (aka perceived trust)<br>*-Privacy concern,*<br>*-Perceived susceptibility* (T)*	In cultures high in collectivism, there is a lower impact of privacy concerns on attitude towards CTAs.	1) Individuals are willing to share their personal information on CTAs due to the benefit to the society.	1) CTAs should not be the only tool for fighting COVID-19, but one of several preventive and detective measures such as manual tracing, decontamination, hygiene, and social distancing. 2) Privacy statements regarding CTA need to be transparent and informative. They should specify exactly when the collected data will be accessed and how and what it will be used for.
Walrave et al. [2]	April 2020	Descriptive	Belgium (n=1500, age=18+)	QT (SEM: UTAUT)	Intention to use (R^2=39%)	-Perceived usefulness<br>-Facilitating conditions (perceived compatibility)<br>-Social influence<br>-Innovativeness<br>*-Privacy concern*	No age, gender, education, and health condition effect on behavior intention.	1) The strongest predictor of CTA uptake intention is perceived usefulness.<br>2) When governments launch CTAs, personal and social benefits should be emphasized and made clear.	1) Minimize data collection to increase uptake.<br>2) Inform potential users about the data needed by the CTA for it to be effective.<br>3) To minimize the amount of time required to read and assess privacy terms, use a visual presentation to improve comprehension.<br>4) CTAs should state the personalized services and information to be offered.
Altmann et al. [3]	April 2020	Descriptive	France, Germany, Italy, UK, and US	Mixed	Intention to install	-Automatic installation by mobile phone providers	Germany and United States were relatively less	1) Overall, there is strong public support for CTAs, which is a necessary condition for their viability.	1) An opt-out regime is more likely to result in higher effective installation rates than an opt-in regime, e.g., by reducing the

			(n=5995, age=18+)			-Voluntary installation<br>*-Privacy concern*<br>*-Cybersecurity concern*<br>*-Lack of trust in government*	supportive compared with the other countries.	74.8% of subjects across all countries said they would probably or definitely download the CTA if available. 2) 60% of participants said they would consent to making their deidentified data available to research.	negative effects of procrastination or unawareness.<br>2) Governments should consider delegating digital contact tracing to highly reputable and transparent public health authorities over whom they have little to no control.
Abuham<br><br>mad et al.<br><br>[4]	July 2020	Descripti ve	Jordan (n=1654, age=18+)	QT	Intention<br><br>to use	-Perceived benefit<br>-Voluntariness<br>*-Privacy concern*<br>*-Accessibility*<br>*-Technical problem*<br>*-Legal problem*<br>*-Participation cost*<br>*-Limited information*	Lower income and better living area increase the acceptance and use of CTAs.	1) 71.6% accepted to use CTAs, with 37.80% being current users. CTAs provide both personal and social benefits. Although CTA acceptance was high, actual use was comparably less. 2) 82.3% of participants concurred that the community should regulate CTAs. 3) The majority of participants agreed that WHO (85.6%) and CTA software companies were most important in terms of having access to the CTA data.	1) Information about CTAs, e.g., objectives, description, how it works, sponsors, potential risks, possible benefits, and voluntariness of use, should be provided.<br>2) Researchers should find ways to inform users about the results of their data analysis.<br>2) To promote public confidence, users should be informed that CTAs work without location data utilization.<br>3) Governments and healthcare providers should intensify efforts to promote actual use of CTAs.
Kaspar [5]	April – May 2020	Descripti ve	Germany (n=406, age=18+)	QT (RM: PMT)	Intention to use (R^2=46%)	-Perceived social distancing (SD) self-efficacy<br>-SD response efficacy<br>-SD response costs<br>-Perceived trust in app providers<br>*-Perceived severity of data misuse*<br>*-Perceived vulnerability to data misuse*<br>*-Perceived trust in other people's SD behavior*	Age and gender do not have a significant effect on intention to use.	1) SD coping appraisals and perceived trust in app providers has a positive effect on intention to use CTAs.<br>2) Participants are more likely to use a CTA than a pure data donation app that provides its users no direct utility.<br>3) Many of the bivariate correlations between independent and dependent variables disappeared in a regression model.	1) CTA stakeholders should promote SD coping skills and recommend practical guidelines to improve users' perceived self-efficacy with regard to compliance.<br>2) CTA stakeholders should foster trust in CTAs by implementing strong data security measures and effectively communicating them to users.

Jonker et al. [6]	April 2020	Descriptive	Netherlands (n=900, age=15+)	QT (Logit model)	Intention to use (based on app version preference)	-Financial reward<br>-Non-financial reward (permission to socialize)<br>-User-controlled data sharing<br>-Privacy protection<br>*-Privacy concern*<br>*-Data security concern*<br>*-Non-ownership of phone*<br>*-Out-of-pocket cost*	1) Older adults and less educated people are less willing to install CTAs.<br>2) Presence of underlying serious health conditions increases CTA adoption.	1) Over 50% of the respondents always chose a CTA of a given specification; about 25% could never be persuaded to choose; and the choice of the remaining 24% depended on the app specifications.<br>2) Overall, subjects preferred CTAs that offer additional benefits such as a small monetary reward of €5 or €10 per month, permission to gather in small groups and free testing after receiving an exposure alert.	2) CTA users should be given control of their data, including the permission to share exposure notifications with authorities and report diagnoses.<br>3) Respondents preferred exposure notifications that specify date and time.<br>3) Governments should implement secure and privacy-preserving CTAs with sufficient realistic features. This has the potential to produce an adoption rate as high as 64% in the Netherlands.
Thomas et al. [7]	May 2020	Official App - COVIDSafe	Australia (n=1500, age=18+)	Mixed	Intention to download app	*-Privacy concern*<br>*-Technical concern*<br>*-Perceived unnecessariness*<br>*-Perceived distrust*<br>*-Doubt about effectiveness*<br>*-Limited app information*	Age positively influences app download.	1) The most frequent reason respondents did not download the COVIDSafe app is privacy concerns (25%), followed by technical problems (24%), app unnecessariness (17%), distrust in the government (11%), doubt about app effectiveness (7%), and need for more information before deciding (5%).<br>2) Only 30% of non-adopters were willing to download the app.	1) CTA sponsors should sensitize potential users that the app would not alert them once they come near an infected person, as 72% of the respondents incorrectly thought this.<br>2) CTA sponsors should sensitize potential users that their personal information would not be shared after the pandemic, as 50% of the study respondents incorrectly thought this.
Cruz et al. [8]	July 2020	Prototype - RISCOVID	Brazil (n1=201, n2=30 , age=18+)	Mixed	Willingness to share location, intention to use app and intention to report diagnosis (based on	-Self-monitoring<br>-Social-location monitoring<br>-Tangible reward<br>*-Privacy concern*	Age and education had no significant effect on willingness to share location.	1) Over 65% of respondents were willing to share their location even without any reward. This percentage increased when users were offered tangible rewards.<br>2) Over 50% of subjects (n = 201) wanted to know how many infected people they had come in contact with,	Participants (n = 201) are more willing to share their locations when they are offered access to the health system (76%) and a tangible reward (71%) than offered no tangible reward (65%). Hence, future CTAs should support some types of tangible incentives to motivate users to adopt and use CTAs.

					a CTA prototype)			have passed through a given location, and the commercial spot with the highest number of infected persons.<br>3) Participants (n =30) perceived the prototype as useful, easy to use and had high intentions to use it.	
Walrave et al. [9]	April 2020	Descriptive	Belgium (n=730, age=18+)	QT (SEM: HBM)	Intention to use (R^2=32%)	-Perceived benefit<br>-IT self-efficacy<br>-Cues to action<br>*-Privacy concern*	Age has a negative effect on perceived susceptibility, benefits and IT self-efficacy, but positive effect on perceived severity.	1) Perceived benefit is the most important predictor of intention to use CTA, followed by IT self-efficacy, perceived barriers, and cues to action (from traditional/social media).<br>2) Perceived severity (H) and perceived susceptibility (H) were not significantly related to intention to use CTA.	1) App designers should emphasize the perceived benefits and decrease perceived barriers such as privacy concerns by assuring potential users that their privacy and data will be protected.<br>2) National media can play a crucial role in improving the uptake of CTAs by informing citizens about their functions, benefits, and use cases, thereby increasing perceived IT self-efficacy.
Velicia-Martin et al. [10]	Prior to July 2020	Descriptive	Public, Social Media (n=482, age=15+)	QT (SEM: TAM/ HBM	Intention to use (R^2=77%)	-Perceived trust<br>-Perceived ease of use<br>-Perceived usefulness<br>-Attitude towards CTA<br>*-Perceived susceptibility (H)*	Age, education and employment moderates at least two of the relations between perceived usefulness/trust/COVID-19 risk/privacy concern and intention to use CTA.	1) Users would be willing to use CTAs if they trust it (especially employed users), value its usefulness, and find it easy to use.<br>2) Among the target population (some of whom are social media), users do not care about privacy in the light of health issues.<br>3) The higher users perceive the risk of COVID-19 and/or their susceptibility, the more likely they are to use CTAs.	1) The governments, who are the major sponsors of CTAs, should emphasize the finding that older people are more likely to be susceptible to COVID-19; this has the potential of increasing adoption rate among the older population.<br>2) Among the target population, the government should emphasize the health benefit (e.g., curbing the spread of the virus and reducing the users' vulnerability) and the ease of using the app more than the privacy concerns.
Trang et al. [11]	April 2020	Descriptive/ Prototype (COV-	Germany (n=518, age mean = 34)	QT (RM)	Intention to install	-Convenience design<br>-Coronavirus infection anxiety<br>-Privacy design	Age, gender and education moderate the	1) Critics respond to the social benefit and privacy design of CTAs in their installation decision.	1) In a heterogeneous population, policy makers should tailor the CTA to those who need to be convinced.

		19 WATCH)				-IT self-efficacy<br>-Social benefit<br>*-Self-benefit appeal*<br>*-Privacy concern*<br>*-Self-social-benefit appeal*	relationship between the predictors and intention to install.	2) For undecided citizens, emphasizing privacy design and social benefits is more likely to be effective than self-benefits, which are counterproductive, even when combined with social-benefit appeal.<br>3) None of the benefit appeals (self, social or combined) is superior in achieving mass acceptance among advocates that make up the majority.	2) If the groups of advocates and undecided are large, policy makers should focus on high privacy and convenience to convince those who need to be convinced.<br>3) In addition, in a heterogeneous population, to achieve mass acceptance, they should emphasize social (altruistic) benefits rather than self-benefits.
Jansen-Kosterink et al. [12]	April 2020	Descriptive	Nether-lands (n=238, age=18+)	Mixed (QT: RM)	Intention to use (R^2=16%)	-Coronavirus infection anxiety<br>-Attitude towards technology<br>*-Privacy concern*	Age has a positive effect on intention to use.	1) The most frequent reason why people want to use the app is the control of the spread of the virus (31% of subjects), followed by gaining insight into the spread of the virus (23%), personal health (13%) and safety (12%).<br>2) The least frequent reason why people want to use the app is fear of COVID-19 (1.4% of subjects), followed by protecting the frail population (4%) and social good (6%).<br>3) The most frequent reason why people do not want to use the app is lack of willingness to share their data with the government for privacy reasons (65%); the second most frequent reason is doubt of perceived usefulness (13%).	1) Governments should find effective ways of increasing the confidence of the citizens in the data and privacy protection mechanisms implemented in CTAs.<br>2) Governments in their awareness campaigns should emphasize the public health benefit of using CTAs to curb the spread of the virus.<br>3) Particularly, government should tailor their campaigns to the younger population more compared to the older population.

Li et al. [13]	November 2020	Descriptive	United States (n=1963, age=18+)	QT	Intention to install (R^2=33%)	-Contact location storage on device<br>-Contact location upload (if user tests positive)<br>-Prosocialness<br>-Perceived COVID-19 risk<br>-Technology readiness<br>*-Privacy concern*<br>*-Data security concern (secondary use of data risk)*	1) Older people, females, and essential workers are less willing to install CTAs.<br>2) Health/ essential workers preferred CTAs with no location tracking.<br>3) Hispanics, high-income earners, frequent public-transit users, and urban residents are more willing to install CTAs.	1) The perceived benefit of CTAs is a more important determinant of the intention to install than the perceived security and privacy risks.<br>2) Users were more willing to install CTAs that collect location data than those that do not due to the additional benefits (e.g., provision of hotspot information and analysis). 3) Unlike app design which has a small effect, individual differences had a large effect on CTA adoption intentions. For example, people with higher prosocialness, higher perceived susceptibility and higher technology readiness are significantly more willing to install CTAs.	1) CTAs should go beyond a one-size-fits-all design. For example, they should support opt-in features (e.g., location data) to allow users who are willing to contribute more data to have access to more useful features such as hotspots, while enabling users who are more concerned about the security and privacy risks to share the minimum amount of data of their choice.<br>2) In addition to serving an educational purpose (e.g., emphasizing the public health benefit), CTAs should provide basic statistics visualizations to help users track their exposure levels and make a better sense of the personal benefits in self-monitoring.

Reference

1. Sharma S, Singh G, Sharma R, et al (2020) Digital Health Innovation: Exploring Adoption of COVID-19 Digital Contact Tracing Apps. IEEE Transactions on Engineering Management. https://doi.org/10.1109/TEM.2020.3019033
2. Walrave M, Waeterloos C, Ponnet K (2020) Ready or Not for Contact Tracing? Investigating the Adoption Intention of COVID-19 Contact-Tracing Technology Using an Extended Unified Theory of Acceptance and Use of Technology Model. Cyberpsychology, Behavior, and Social Networking 1–7. https://doi.org/10.1089/cyber.2020.0483
3. Altmann S, Milsom L, Zillessen H, et al (2020) Acceptability of app-based contact tracing for COVID-19: Cross-country survey study. JMIR mHealth and uHealth 8:. https://doi.org/10.2196/19857
4. Abuhammad S, Khabour OF, Alzoubi KH (2020) Covid-19 contact-tracing technology: Acceptability and ethical issues of use.

Patient Preference and Adherence 14:1639–1647. https://doi.org/10.2147/PPA.S276183

5. Kaspar K (2020) Motivations for social distancing and app use as complementary measures to combat the COVID-19 pandemic: Quantitative survey study. Journal of Medical Internet Research 22:e21613. https://doi.org/10.2196/21613
6. Jonker M, de Bekker-Grob E, Veldwijk J, et al (2020) COVID-19 contact tracing apps: Predicted uptake in the Netherlands based on a discrete choice experiment. JMIR mHealth and uHealth 8:. https://doi.org/10.2196/20741
7. Thomas R, Michaleff ZA, Greenwood H, et al (2020) Concerns and misconceptions about the australian government's COVIDsafe app: Cross-sectional survey study. JMIR Public Health and Surveillance 6:. https://doi.org/10.2196/23081
8. Cruz MM, Oliveira RS, Beltrao APV, et al (2020) Assessing the level of acceptance of a crowdsourcing solution to monitor infectious diseases propagation. 2020 IEEE International Smart Cities Conference, ISC2 2020. https://doi.org/10.1109/ISC251055.2020.9239069
9. Walrave M, Waeterloos C, Ponnet K (2020) Adoption of a contact tracing app for containing COVID-19: A health belief model approach. JMIR Public Health and Surveillance 6:. https://doi.org/10.2196/20572
10. Velicia-Martin F, Cabrera-Sanchez JP, Gil-Cordero E, Palos-Sanchez PR (2021) Researching COVID-19 tracing app acceptance: incorporating theory from the technological acceptance model. PeerJ Computer Science 7:1–20. https://doi.org/10.7717/peerj-cs.316
11. Trang S, Trenz M, Weiger WH, et al (2020) One app to trace them all? Examining app specifications for mass acceptance of contact-tracing apps. European Journal of Information Systems 29:415–428. https://doi.org/10.1080/0960085X.2020.1784046
12. Jansen-Kosterink SM, Hurmuz M, den Ouden M, van Velsen L (2020) Predictors to use mobile apps for monitoring COVID-19 symptoms and contact tracing: A survey among Dutch citizens. medRxiv. https://doi.org/10.1101/2020.06.02.20113423
13. Li T, Cobb C, Yang J, et al (2020) What makes people install a COVID-19 contact-tracing spp? Understanding the influence of app design and individual difference on contact-tracing app adoption intention. arXiv
