# Supplementary material for: Factors Influencing the Adoption of Contact Tracing Applications: Systematic Review and Recommendations
Source: Front Digit Health. 2022 May 3;4:862466. doi: 10.3389/fdgth.2022.862466 (PMC9110790; doi:10.3389/fdgth.2022.862466)
Supplement: Supplementary file 2 [file Data_Sheet_2.pdf]

Supplementary File 2: Factors Influencing the Adoption of Contact Tracing Applications: Systematic Review and Recommendations

Kiemute Oyibo; Kirti Sundar Sahu; Arlene Oetomo; and Plinio Pelegrini Morita

Definition of Key Terms and CTA Factors

Supplementary Table 2 provides a definition of the key factors that influence CTA adoption, which we elicited from the 13 included articles.

Supplementary Table 2. Definitions of factors of CTA adoption. T: Technology related, H: Health related, SD: Social Distancing.

**Factor**	**Definition**
Perceived risk (T)	It is the subjective assessment of the vulnerability to a technological threat (e.g., a data breach) and the probability of being harmed to a certain extent. It is made up of three dimensions: perceived vulnerability, perceived likelihood and perceived severity (1).
Perceived vulnerability (T)	It is the subjective assessment of one's vulnerability to a technological threat.
Perceived likelihood (T)	It is the subjective assessment of the probability of being harmed by a technological threat.
Perceived susceptibility (T)	It is the subjective assessment of one's vulnerability and likelihood to be harmed by a technological threat. It is made up of two dimensions: perceived vulnerability and perceived likelihood.
Perceived severity (T)	It is the subjective assessment of the extent of the harm caused by a technological threat.
Perceived risk (H)	It is the subjective assessment of the vulnerability to a health condition (e.g., a disease) and the probability of being harmed to a certain extent. It is made up of three dimensions: perceived vulnerability, perceived likelihood and perceived severity (1).
Perceived vulnerability (H)	It is the subjective assessment of one's vulnerability to a health condition.
Perceived likelihood (H)	It is the subjective assessment of the probability of being harmed by a health condition.
Perceived susceptibility (H)	It is the subjective assessment of one's vulnerability and likelihood to be harmed by a health condition. It is made up of two dimensions: perceived vulnerability and perceived likelihood.
Perceived severity (H)	It is the subjective assessment of the extent of the harm caused by a health condition.
Coronavirus (infection) anxiety	It is the worry about and/or fear of a COVID-19 infection.
Perceived usefulness	The degree to which a user believes using a CTA will help curb the spread of the coronavirus (2). It is also known perceived benefit (or benefit appeal (3)) and a determinant of behavior in the Health Belief Model (HBM) (4) (5).
Personal benefit	It is the personal benefit a user derives from using a CTA, e.g., protection of personal health (3).

Social benefit	It is the benefit the society or public derives from a user's using a CTA, e.g., protection of public health (3). People who want to use the app partly or wholly due its social benefit are described as prosocial.
Self-Social benefit	It is the benefit the individual and society derive from a user's using a CTA, e.g., protection of personal and public health (3).
Prosocialness	It is the tendency to carry out voluntary behaviors and/or actions to help or benefit other people than self. Although it is related to altruism, prosocialness refers to the activities carried out to help and support others, while altruism is the motivation to help other people out of pure regard for their need (6).
Perceived ease of use	The degree to which a user believes using a CTA will be free of effort and difficulty (2).
Privacy self-efficacy	An individual's judgment and confidence in themselves to manage privacy issues (7).
Perceived compatibility	It is the belief that one has the necessary resources and knowledge to use a new app, e.g., because it is consistent with previous ones (8).
Privacy design	It is the design of CTAs to reduce privacy concerns, e.g., implementing Bluetooth rather than GPS tracing, decentralized rather than centralized processing of data, restricted rather than extended data usage, etc. (3).
Convenience design	It is the design of CTAs to reduce privacy concerns, e.g., implementing low rather than high battery depletion, automatic rather than regular manual updates etc. (3).
Innovativeness	It is the tendency to be one of the first to adopt new technologies at inception and inform/advise others about them (8).
Technical concern	Technical issues that may be a barrier to the adoption of CTAs, e.g., non-ownership of smartphones, low phone storage space, etc. (9)(10).
Attitude towards CTA	It is an individual's thinking or feeling about CTAs which can influence their installing and/or using them (7)(11)(12).
Subjective norm	The beliefs of an individual's significant others (e.g., friend, family, etc.) regarding the individual's adopting of CTAs (7).
SD self-efficacy	It is the belief in one's ability to social distance in public spaces (13).
SD response efficacy	It is the belief in the effectiveness of social distancing behavior in public spaces, e.g., protecting individuals from COVID-19 (13).
SD response cost	It is the cost (e.g., the exhaustive nature) of maintaining social distance in public spaces (13).
Perceived trust in others' SDB	It is the belief that other people will social distance as well (13).
Perceived social safety	It is the belief that one is safe from COVID-19 infection in large groups (14).

Clarification of Perceived Risk and its Dimensions

The constructs (perceived risk, perceived susceptibility, perceived vulnerability, and perceived severity) are often confused in the literature on health and technological systems and even used interchangeably sometimes. For example, Mimiaga et al. (15) defined perceived susceptibility as "*perceive[d] vulnerability to [a] specific disease [or threat]*" (15). This means that perceived susceptibility is deemed the same as perceived vulnerability by Mimiaga et al. Moreover, Sharma et al. (7) in the current review stated, "*Perceived vulnerability is an individual's evaluation of possibly encountering a threat. An individual's perception of the negative consequences of sharing personal information is described as vulnerability*" (p. 4). The first sentence in Sharma et al.'s explanation of perceived vulnerability focuses on the *likelihood* or *possibility* of encountering a threat, while the second sentence focuses on the *consequence* of the threat. It is noteworthy that Sharma's explanation of perceived vulnerability is similar to Molina's definition of perceived risk: "evaluations of the *probability* as well as the *consequences* of an uncertain outcome" (p. 1690). The similarity in Sharma et al.'s explanation of perceived vulnerability and Molina's definition of perceived risk is an indication of poor understanding of perceived risk and its dimensions.

Secondly, Sharma et al. (7) defined perceived severity as "*the negative consequences perceived by individuals as a result of security threats*" (p. 3) instead of "*the extent to which the negative consequences are perceived by the individual.*" Rather than capturing the *severity* of the negative consequences of using technology, Sharma et al.'s definition of perceived severity focuses on the negative consequences only, which is similar to their initial explanation of perceived vulnerability: "*An individual's perception of the negative consequences of sharing personal information*" (p. 4) (7). Again, this confusion in definitions and explanations is an indication of poor understanding of the dimensions of perceived risks by authors. Similarly, Mimiaga et al.'s (15) definition of perceived severity in a question form, "*does the individual perceive that getting the disease has negative consequences*?," reflects the poor understanding of the dimensions of perceived risks and their definitions by auhors in the extant the literature. However, unlike their definition, Sharma et al.'s operationalization of perceived severity appropriately reflects the term "severity." For example, the first item as shown reads, "*If my information privacy is invaded, it would be severe*;" the second item reads, "*If my information privacy is invaded, it would be serious*;" and the third item reads, "*If my information privacy is invaded, it would be significant*." Hence, there is a need for a clarification of the definitions of perceived risk and its dimensions.

Although Molina (1) states that perceived risk is composed of three dimensions (perceived likelihood, perceived susceptibility, and perceived severity), her definition of "perceived risk" does not explicitly cover the severity dimension, which she defines as "the *extent* of harm a hazard would cause." Moreover, her conceptualization of the dimensionality of perceived risk does not explicitly include perceived vulnerability, which is often measured in user studies of health and technological systems (16)(13) (see Supplementary Table 1). Due to the confusion in conceptualizing, defining, and differentiating perceived risk and its dimensions, we provide a clarification to make the differentiation of all five constructs (perceived

risk, perceived likelihood, perceived susceptibility, perceived severity, and perceived vulnerability) easier to grasp and operationalize. The differentiation will help health and technological system researchers, who employ them in their work, to communicate knowledge with a common understanding and usage of the terms. This will make the synthesis of findings in systematic reviews such as ours to be easier, as all researchers have a common understanding of perceived risk, its dimensions, and their operationalization. Particularly, the clarification will prevent inappropriate definitions of the risk-related constructs, which may culminate in inappropriate operationalization.

According to the Oxford Lexico dictionary (17), vulnerability is defined as "the quality or state of *being exposed* to the possibility of being attacked or harmed." Moreover, susceptibility is defined as "the state or fact of being *likely* or *liable* to be influenced or harmed by a particular thing" (18). This definition of susceptibility can be paraphrased as "the *likelihood* of being harmed by a certain threat and/or one's *liability* to the threat." Hence, as shown in Supplementary Figure 1, we conceive perceived susceptibility as having two dimensions (*perceived likelihood* and *perceived vulnerability*) and define it accordingly, with "liability" regarded as equivalent to "vulnerability" (see Supplementary Table 2). Furthermore, based on Molina's (1) work, we conceived perceived risk as comprising two dimensions (perceived susceptibility and perceived severity). Overall, this means perceived risk comprises three dimensions altogether: perceived vulnerability, perceived likelihood and perceived severity, as propounded by Molina (1). Hence, based on this new understanding of perceived risk, we define perceived risk in a broader sense that encompasses its three dimensions thus: "*the subjective assessment of one's vulnerability to harm and the probability and extent of being harmed*." This conceptual framework for delineating the components of perceived risk (Supplementary Figure 1) is consistent with Malmadal and Roislien's (19) definition of risk perception, which, unlike Molina's definition, does capture the severity dimension: "*the judgement that people make about the characteristics and severity of a risk*." The first component of Malmadal and Roislien's definition ("characteristics of a risk") can be regarded as perceived susceptibility (i.e., perceived vulnerability and perceived likelihood) in the conceptual framework of perceived risk shown in Supplementary Figure 1.

Hence, in the context of health and technological threats, as shown in Supplementary Table 2, perceived risk can be defined as "*the subjective assessment of the vulnerability to a health condition (technological threat) and the probability and extent of being harmed*." In line with this definition of perceived risk illustrated in Supplementary Figure 1, we renamed some of the related constructs in the reviewed articles appropriately for easy synthesis of the findings of the systematic review. For example, we renamed "perceived risk" in Velicia-Martin et al.'s (11) study as "perceived susceptibility" in this systematic review because the items operationalizing the former construct are only concerned with vulnerability and likelihood. For instance, the item, "*I feel that we may be vulnerable to COVID-19 infection*," relates to perceived vulnerability. Moreover, the item, "*I think the chances of us getting infected with COVID-19 are very high*," relates to perceived likelihood. Similarly, we renamed "perceived vulnerability" in Sharma et al.'s (7) study as

"perceived susceptibility" because the three items operationalizing the former construct are concerned with vulnerability and likelihood. For instance, as shown in Supplementary Table 3, the item, "*My information privacy is at risk of being invaded*," relates to perceived vulnerability. Moreover, the items, "*it is likely that my information privacy will be invaded*," and "*it is possible that my information privacy will be invaded*," relate to perceived likelihood.

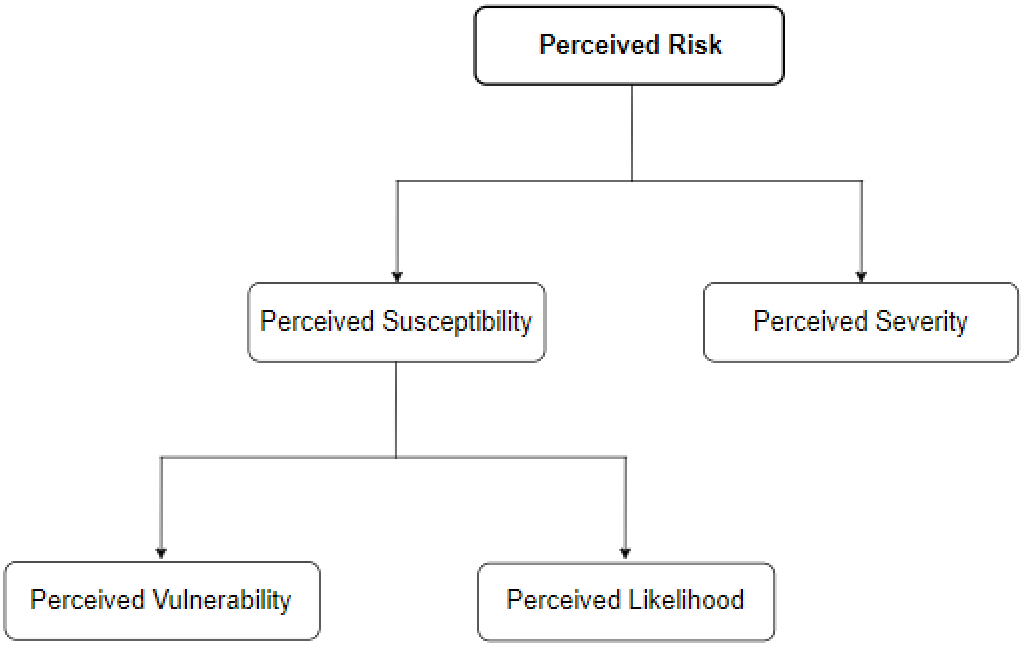

Supplementary Figure 1. A conceptual framework for understanding perceived risk and its dimensions in the context of health and technological threats.

Supplementary Table 3. Example operationalization of the dimensions of perceived risk in the context of technological systems that deal with information privacy "*": items that were originally listed as part of perceived vulnerability scale in Sharma et al.'s (7) study.

**Dimension**	**Adapted Example Items Operationalizing Dimension**
Perceived vulnerability (7)(11)(20)	(1) My information privacy is at risk of being invaded.<br>(2) I feel vulnerable to information privacy invasion.<br>(3) I feel my information privacy is subject to cyberattacks.
Perceived likelihood (7)(11)	(1) It is possible that my information privacy will be invaded.*<br>(2) It is likely that my information privacy will be invaded.*<br>(3) I think the chances of my information privacy being invaded are very high.
Perceived severity (7)	(1) If my information privacy is invaded, it would be severe.<br>(2) If my information privacy is invaded, it would be serious.<br>(3) If my information privacy is invaded, it would be significant.

Based on the diagrammatic framework shown in Supplementary Figure 1, in empirical studies, in addition to testing the reliability of a constructs using metrics such as the Cronbach's alpha and McDonald's omega (21)(22), the construct can be

said to have been fully measured if it meets two conditions. The first condition is the operationalization of the construct encompasses items from all of its dimensions. The second condition is that the items that passed the internal consistency reliability test encompass items from all of its dimensions. For example, "perceived risk" can be said to have been measured if the items touch on its three dimensions (perceived vulnerability, perceived likelihood, and perceived severity). Similarly, "perceived susceptibility" can be said to have been measured if the items touch on its two dimensions (perceived vulnerability and perceived likelihood). Based on this guideline, if a researcher measures, say, perceived risk by picking items from the three dimensions shown in Supplementary Table 3, and the reliability test turns out that only a subset of the items relating to two of the dimensions (e.g., perceived vulnerability and perceived likelihood) passed the reliability test, then the researcher should report that "perceived susceptibility" and not "perceived risk" was measured. However, if the items that passed the test relates to one of the dimensions of perceived susceptibility (perceived vulnerability or perceived likelihood) and perceived severity, then the research may report that perceived risk was measured with the caveat that one of the dimensions of perceived susceptibility or perceived risk did not pass the reliability test.

References

1. Molina KM, Molina KM, Goltz HH, Kowalkouski MA, Hart SL, Latini D, et al. Racial Inequality in Economic and Social Well-Being. In: Encyclopedia of Behavioral Medicine. 2013. p. 1611–2.
2. Davis FD. Perceived Usefulness , Perceived Ease of Use , and User Acceptance of Information Technology. 1989;319–40.
3. Trang S, Trenz M, Weiger WH, Tarafdar M, Cheung CMK. One app to trace them all? Examining app specifications for mass acceptance of contact-tracing apps. European Journal of Information Systems. 2020;29(4):415–28.
4. Capapé C, Seck AA, Quignard JP. The Health Belief Model: A Decade Later. Health Education Quarterly. 1999;23(3):259–71.
5. Walrave M, Waeterloos C, Ponnet K. Adoption of a contact tracing app for containing COVID-19: A health belief model approach. JMIR Public Health and Surveillance. 2020;6(3).
6. Knickerbocker RL. Prosocial Behavior [Internet]. Available from: https://www.learningtogive.org/resources/prosocial-behavior
7. Sharma S, Singh G, Sharma R, Jones P, Kraus S, Dwivedi YK. Digital Health Innovation: Exploring Adoption of COVID-19 Digital Contact Tracing Apps. IEEE Transactions on Engineering Management. 2020;
8. Walrave M, Waeterloos C, Ponnet K. Ready or Not for Contact Tracing? Investigating the Adoption Intention of COVID-19 Contact-Tracing Technology Using an Extended Unified Theory of Acceptance and Use of Technology Model. Cyberpsychology, Behavior, and Social Networking. 2020;1–7.
9. Abuhammad S, Khabour OF, Alzoubi KH. Covid-19 contact-tracing technology: Acceptability and ethical issues of use. Patient Preference and Adherence. 2020;14:1639–47.
10. Thomas R, Michaleff ZA, Greenwood H, Abukmail E, Glasziou P. Concerns and

misconceptions about the australian government's COVIDsafe app: Cross-sectional survey study. JMIR Public Health and Surveillance. 2020;6(4).
11. Velicia-Martin F, Cabrera-Sanchez JP, Gil-Cordero E, Palos-Sanchez PR. Researching COVID-19 tracing app acceptance: incorporating theory from the technological acceptance model. PeerJ Computer Science. 2021;7:1–20.
12. Jansen-Kosterink SM, Hurmuz M, den Ouden M, van Velsen L. Predictors to use mobile apps for monitoring COVID-19 symptoms and contact tracing: A survey among Dutch citizens. medRxiv. 2020;
13. Kaspar K. Motivations for social distancing and app use as complementary measures to combat the COVID-19 pandemic: Quantitative survey study. Journal of Medical Internet Research. 2020;22(8):e21613.
14. Jonker M, de Bekker-Grob E, Veldwijk J, Goossens L, Bour S, Mölken MR Van. COVID-19 contact tracing apps: Predicted uptake in the Netherlands based on a discrete choice experiment. JMIR mHealth and uHealth. 2020;8(10).
15. Mimiaga MJ, Reisner SL, Reilly L, Soroudi N, Safren SA. Individual interventions. In: HIV Prevention. Academic Press; 2009. p. 203–39.
16. Altmann S, Milsom L, Zillessen H, Blasone R, Gerdon F, Bach R, et al. Acceptability of app-based contact tracing for COVID-19: Cross-country survey study. JMIR mHealth and uHealth. 2020;8(8).
17. Vulnerability [Internet]. Oxford Lexico. 2022 [cited 2022 Jan 2]. Available from: https://www.lexico.com/definition/vulnerability
18. Susceptibility [Internet]. Oxford Lexico. 2022 [cited 2022 Jan 2]. Available from: https://www.lexico.com/definition/susceptibility
19. Malmadal B, Roislien H. Cybersecurity risk perception. Norwegian Centre for Information Security. 2016;1–22.
20. Schaffer DR, Debb SM. Validation of the Online Security Behaviors and Beliefs Questionnaire with College Students in the United States. Cyberpsychology, Behavior, and Social Networking. 2019;22(12):766–70.
21. Dunn TJ, Baguley T, Brunsden V. From alpha to omega: A practical solution to the pervasive problem of internal consistency estimation. British Journal of Psychology. 2014;105(3):399–412.
22. Oyibo K, Ali YS, Vassileva J. An empirical analysis of the perception of mobile website interfaces and the influence of culture. In: Proceedings of Personalized Persuasive Technology Workshop [Internet]. Salzburg, Austria; 2016. p. 44–56. Available from: http://ceur-ws.org
